# An injectable magnesium-loaded hydrogel releases hydrogen to promote osteoporotic bone repair via ROS scavenging and immunomodulation

**DOI:** 10.7150/thno.97412

**Published:** 2024-06-17

**Authors:** Hang Zhou, Zhongyuan He, Youde Cao, Lei Chu, Bing Liang, Kexiao Yu, Zhongliang Deng

**Affiliations:** 1 Department of Orthopaedics, the Second Affiliated Hospital of Chongqing Medical University, 76 Linjiang Road, Yuzhong Distinct, Chongqing, 400010, P. R. China.; 2 Department of Pathology from College of Basic Medicine, and Molecular Medicine Diagnostic & Testing Center, and Department of Clinical Pathology Laboratory of Pathology Diagnostic Center, Chongqing Medical University, 1 Yixueyuan Road, Yuzhong Distinct, Chongqing 400016, P. R. China.; 3 Department of Orthopedics, Chongqing Traditional Chinese Medicine Hospital, The First Affiliated Hospital of Chongqing College of Traditional Chinese Medicine, No. 6 Panxi Seventh Branch Road, Jiangbei District, Chongqing 400021, P. R. China.; 4 Department of Ultrasound & Chongqing Key Laboratory of Ultrasound Molecular Imaging, the Second Affiliated Hospital of Chongqing Medical University, Chongqing 400010, P. R. China.

**Keywords:** osteoporotic bone defect, ROS scavenging, hydrogen, magnesium-based hydrogel, immunomodulation

## Abstract

**Background:** The repair of osteoporotic bone defects remains challenging due to excessive reactive oxygen species (ROS), persistent inflammation, and an imbalance between osteogenesis and osteoclastogenesis.

**Methods:** Here, an injectable H_2_-releasing hydrogel (magnesium@polyethylene glycol-poly(lactic-co-glycolic acid), Mg@PEG-PLGA) was developed to remodel the challenging bone environment and accelerate the repair of osteoporotic bone defects.

**Results:** This Mg@PEG-PLGA gel shows excellent injectability, shape adaptability, and phase-transition ability, can fill irregular bone defect areas via minimally invasive injection, and can transform into a porous scaffold *in situ* to provide mechanical support. With the appropriate release of H_2_ and magnesium ions, the 2Mg@PEG-PLGA gel (loaded with 2 mg of Mg) displayed significant immunomodulatory effects through reducing intracellular ROS, guiding macrophage polarization toward the M2 phenotype, and inhibiting the IκB/NF-κB signaling pathway. Moreover, *in vitro* experiments showed that the 2Mg@PEG-PLGA gel inhibited osteoclastogenesis while promoting osteogenesis. Most notably, in animal experiments, the 2Mg@PEG-PLGA gel significantly promoted the repair of osteoporotic bone defects *in vivo* by scavenging ROS and inhibiting inflammation and osteoclastogenesis.

**Conclusions:** Overall, our study provides critical insight into the design and development of H_2_-releasing magnesium-based hydrogels as potential implants for repairing osteoporotic bone defects.

## Introduction

Osteoporosis, a systemic disorder affecting bone metabolism, is characterized by diminished bone mass and deterioration of bone architecture, leading to an elevated risk of brittle fractures and bone defects 1, 2. In the treatment of osteoporotic bone defects, anti-osteoporosis medications, such as bisphosphonates, calcitonin, and teriparatide, are often used as adjunctive therapies alongside surgery to facilitate new bone formation and decrease the risk of refractures 3. Nevertheless, these medications face challenges in reaching the defect area and promoting local bone regeneration directly. Bone grafting, including autologous bone, allograft bone, and artificial bone grafting, is an effective therapeutic approach for treating osteoporotic bone defects, but it is often limited by supply limitations, donor-site morbidity, risks of immunogenicity and disease transfer 4. Traditionally, bioinert materials such as metals and polymethylmethacrylate (PMMA) have been commonly utilized for internal fixation and osteoporotic vertebral compression fractures in clinical practice. However, these materials without bioactivity have poor bone integration efficacy and may lead to stress-shielding effects, resulting in implant loosening or even failure 5, 6. In addition, conventional bone repair biomaterials often fail to achieve the desired clinical outcomes in patients with osteoporosis due to the irregular defect shape, impaired osteoblast activity and bone regeneration ability 7, 8. Hence, novel approaches are urgently needed to avoid these adverse issues and to treat osteoporotic bone defects.

Recent research has collectively indicated that excessive amounts of reactive oxygen species (ROS) in the osteoporotic bone microenvironment significantly hinder bone regeneration 9, 10. In particular, the hydroxyl radical ^•^OH, which has the strongest cytotoxicity, can react indiscriminately with nucleic acids, lipids and proteins, leading to an abnormal inflammatory storm and inhibiting the osteogenesis of bone marrow mesenchymal stem cells 11, 12. To date, numerous antioxidants have been utilized in the development of biomaterials to mitigate inflammation and facilitate osteoporotic bone repair by scavenging ROS. Examples include synthetic nanoenzymes and metallic oxides such as MnO_2_ and CeO_2_ 13, 14. However, the therapeutic effectiveness of these antioxidant agents has been compromised by their limited intratissue diffusion. Furthermore, current ROS-scavenging strategies inadvertently eliminate physiologically beneficial ROS, which play crucial roles in signaling regulation 15. Another concern is the safety and biocompatibility of biomaterials incorporating trace elements. These persistent issues highlight the need for innovative approaches that not only effectively target ROS in osteoporotic bone defects but also preserve the delicate balance of beneficial ROS for physiological functions.

Hydrogen (H_2_), which is emerging as a novel therapeutic agent, has demonstrated exceptional antioxidative and anti-inflammatory properties and holds significant promise for treating ROS-related diseases 16-18. Its superior tissue and cell membrane permeability enable effective intratissue diffusion and targeting of organelles, including mitochondria and nuclei—the primary sources of ROS 19. H_2_ can be instantly delivered to relieve oxidative stress by reacting with and detoxifying highly cytotoxic •OH without disrupting normal metabolic oxidation or cell signaling systems. This ability effectively suppresses inflammation and promotes tissue repair 18. In addition to its therapeutic potential, hydrogen, as an endogenous inert gas, has been proven to be highly safe and biocompatible when administered through various methods, such as inhalation, drinking, intravenous or intraperitoneal injection, and *in situ* injection 18, 20, 21. For example, Chen et al. reported that the consumption of hydrogenated water significantly prevents osteopenia in ovariectomized rats with increased bone mineral content and bone mineral density 22. However, achieving satisfactory efficacy has been challenging due to the short retention time of H_2_ and its extremely low solubility in body fluids. Recent developments in biomaterials aim to address this challenge by facilitating *in situ* H_2_ delivery for the treatment of inflammatory diseases (e.g., rheumatoid arthritis and atherosclerosis) and tissue repair (e.g., bone defects and diabetic wounds) 16, 17, 23, 24. For instance, He et al. designed a H_2_-releasing Bioglass scaffold using electrosprayed polyhydroxyalkanoate-encapsulated CaSi_2_ nanoparticles 16. Their findings demonstrated that the scaffold achieved a local H_2_ supply, effectively remodeling the senescent microenvironment and promoting the healing of critical-size bone defects in an aged mouse model. In summary, molecular hydrogen has significant potential for promoting bone defect repair by ameliorating the harsh microenvironment. However, to our knowledge, no study has reported H_2_-releasing biomaterials specifically for osteoporotic bone defects.

Magnesium (Mg)-based implants have garnered widespread attention for bone tissue repair owing to their outstanding biocompatibility, osteoinductive properties, and biodegradability 25, 26. Previous research has indicated that Mg^2+^ ions within a specific concentration range foster the creation of a pro-osteogenic and anti-osteoclastic immune microenvironment in osteoporosis, highlighting the great potential of Mg-based implants for treating osteoporotic bone defects 25, 27. Recently, researchers have explored the hydrogen generation resulting from the reaction between magnesium particles and H_2_O for ROS scavenging and anti-inflammatory effects 23, 28. For instance, magnesium microparticles coated with hyaluronic acid (Mg-HA) were manufactured and intra-articularly injected for rheumatoid arthritis therapy. Due to the small pores of the HA coating, the Mg-HA microparticles achieved prolonged and sustained H_2_ bubble generation and demonstrated remarkable efficacy in ameliorating joint damage and suppressing the overall severity of arthritis 23. However, these Mg-loaded microspheres cannot provide essential structural support for bone defect sites because of their powder form. In response to this challenge, biodegradable polymer-based injectable solutions known as "*in situ* forming implants" (ISFIs) have gained prominence due to their superior injectability, shape adaptability, and biodegradability 29, 30. After injection at the target site, the ISFIs solidify and transform into scaffolds *in situ*, facilitating drug delivery and tissue regeneration. This approach offers an efficient and minimally invasive strategy for reconstructing osteoporotic bone defects. In our previous work, an injectable phase-transform poly(lactic-co-glycolic acid) (PLGA)/1-methyl-2-pyrrolidinone (NMP) solution (PLGA hydrogel), an FDA-approved ISFI, was developed and applied as a porous biomimetic scaffold for drug delivery and tissue ingrowth. This PLGA hydrogel demonstrated satisfactory efficacy in reconstructing critical-size skull defects and irregular tumorous bone defects 31-33. Importantly, the desired release behavior of drugs and Mg ions was achieved by carefully tuning the loading contents.

**Scheme 1:**
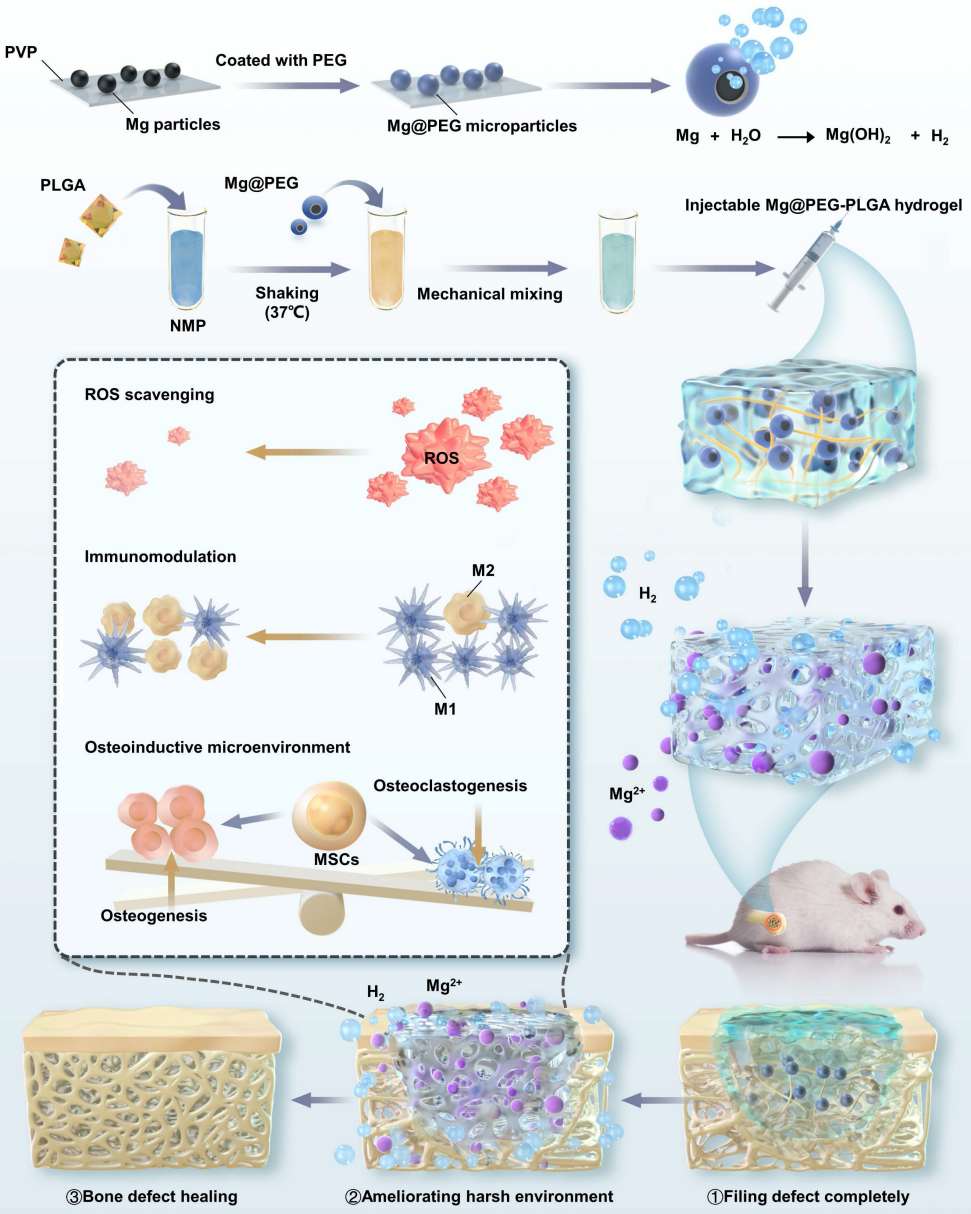
Schematic illustration of the mechanism by which the injectable Mg@PEG-PLGA hydrogel effectively promoted osteoporotic bone regeneration.

In this study, we engineered a Mg-containing PLGA hydrogel (Mg@polyethylene glycol (PEG)-PLGA) for the local delivery of H_2_ and Mg^2+^ ions, which offered several advantages for efficiently remodeling the challenging bone environment and promoting the repair of osteoporotic bone defects (**Scheme 1**). First, the outstanding injectability and compliance of the Mg@PEG-PLGA gel facilitate the minimally invasive filling of bone defects, eliminating the need for additional open surgery. This advantage is particularly beneficial for patients with irregular, deep, or enclosed defects. Second, the Mg@PEG microspheres, which feature an asymmetric coating, react with H_2_O to release H_2_ within the interconnective pores of the Mg@PEG-PLGA gel. This *in situ* reaction increases the porosity of the scaffold, facilitating nutrient/oxygen diffusion and tissue ingrowth. Third, the 2Mg@PEG-PLGA gel (loaded with 2 mg of Mg), which was confirmed to have the desired elastic modulus, porosity, and cytocompatibility, exhibited effective ROS scavenging and anti-inflammatory effects *in vitro* through the suitable release of H_2_ and Mg^2+^ ions. This capability holds promise for ameliorating the harsh bone environment in patients with osteoporosis. Moreover, *in vitro* experiments demonstrated that the 2Mg@PEG-PLGA gel not only inhibited osteoclastogenesis but also promoted osteogenesis. Remarkably, the therapeutic efficacy of the 2Mg@PEG-PLGA gel was confirmed in a rat model of osteoporotic bone defects, in which the bone volume fraction was more than 2-fold greater than that in the control group. Furthermore, RNA-sequencing analysis confirmed the anti-inflammatory and antiosteoclastogenic effects of the 2Mg@PEG-PLGA gel *in vivo*, suggesting that its therapeutic effects may be associated with inhibition of the TNF-α signaling pathway. In summary, the H_2_-releasing Mg@PEG-PLGA gel provides a novel strategy for facilitating osteoporotic bone defect regeneration through ROS scavenging and immunomodulation.

## Results and Discussion

### Synthesis and characterization of Mg@PEG-PLGA hydrogels

**Figure 1:**
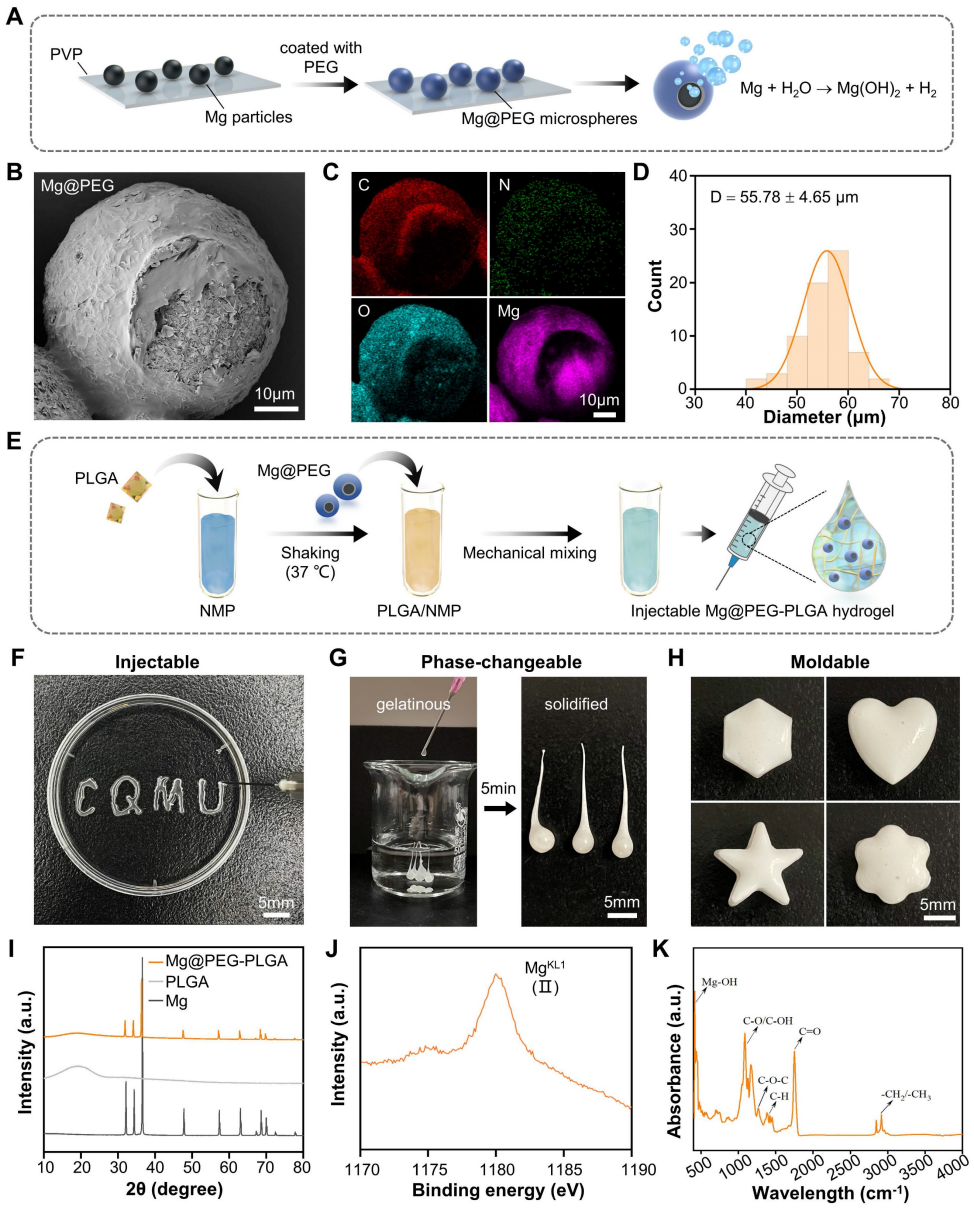
** Synthesis and characteristics of the Mg@PEG-PLGA hydrogel.** (A) Schematic diagram of the preparation of Mg@PEG microspheres. (B) SEM image of Mg@PEG microspheres and the corresponding (C) elemental mapping of C, O, N and Mg. (D) Size distribution of the Mg@PEG microspheres. (E) Schematic diagram of the preparation of the Mg@PEG-PLGA hydrogel. Digital images of the (F) injectability, (G) phase change ability and (H) moldability of the Mg@PEG-PLGA hydrogel. (I) XRD patterns of Mg particles, PLGA and the Mg@PEG-PLGA gel. (J) Mg^KL1^ XPS spectrum of the solidified Mg@PEG-PLGA gel. (K) FTIR spectra of solidified Mg@PEG-PLGA gel.

Compared to implants with prefabricated shapes, injectable shape-forming implants (ISFIs) offer advantages in the treatment of bone defects. They can be delivered via minimally invasive injection and provide mechanical support after undergoing a phase transition 33, 34. In this study, we developed an injectable Mg-based hydrogel by loading a PLGA hydrogel (PLGA/NMP) with PEG-coated Mg microparticles for repairing osteoporotic bone defects. As depicted in **Figure 1A**, Mg@PEG microspheres with an asymmetric structure were prepared by coating Mg particles with biodegradable PEG using an embedding method 23, 28. According to the SEM **(Figure 1B)** and EDS **(Figure 1C** and **Figure S1)** results, the prepared Mg@PEG microspheres exhibited a larger diameter (55.78 ± 4.65 μm)** (Figure 1D)** than did the Mg microparticles (49.49 ± 5.14 μm)** (Figure S2)**. Notably, the asymmetric coating of Mg@PEG microspheres, featuring a small opening (~15 μm) on one side of the structure, served as a Mg-H_2_O reaction interface for the prolonged and controlled release of H_2_ 23, 28. Thermogravimetric analysis was also conducted to determine the actual amount of Mg incorporated into the Mg@PEG microspheres, and the results revealed a Mg content of ~94.6 wt % **(Figure S3)**. Subsequently, the prepared Mg@PEG microparticles were added to the PLGA/NMP solution to form Mg@PEG-PLGA hydrogels (**Figure 1E**) with varying concentrations of Mg particles (0.25, 0.5, 1, 2, and 4 mg/mL), which displayed satisfactory injectability** (Figure 1F)**, phase transformation ability **(Figure 1G)**, and shape adaptability** (Figure 1H)**. The good adhesion of biomaterials to bone defect sites is essential for safe and efficient bone regeneration. The solidified Mg@PEG-PLGA hydrogel in the femoral condylar defect was shown to be firmly bonded to the defect site without displacement or deformation during the rotation test (**Figure S4**). These findings suggest that the injectable Mg@PEG-PLGA hydrogel, when applied *in vivo*, could fill irregular bone defects in a minimally invasive manner, preserving as much surrounding bone tissue as possible—a particularly important consideration for elderly patients with osteoporosis. The XRD analysis results** (Figure 1I)** indicated that the crystalline structure of the Mg microparticles remained unchanged during the synthesis of Mg@PEG-PLGA, preserving the ability to generate H_2_. Previous research has shown that PLGA hydrogel systems can transform into porous scaffolds *in situ* through a liquid-to-solid phase transition, forming an interconnected pore structure with water penetration 31-33. The cross-section of solidified Mg@PEG-PLGA revealed a porous scaffold-like structure within the hydrogel, which was conducive to nutrient/oxygen diffusion and cell ingrowth when implanted *in vivo* 35 (**Figure S5A**). XPS** (Figure S6** and** Figure 1J)** and FTIR **(Figure 1K)** spectra suggested that Mg^2+^ ions and Mg(OH)_2_ formed within the solidified Mg@PEG-PLGA, indicating that some of the incorporated Mg particles reacted with H_2_O during the phase-transition process. Moreover, elemental mapping** (Figure S5B)** demonstrated that these Mg-containing particles were evenly distributed throughout the solidified Mg@PEG-PLGA gel, enabling stable Mg ion release during degradation.

After solidification, the mechanical properties of the Mg@PEG-PLGA hydrogels with different Mg microparticle concentrations were investigated **(Figure S7)**. The results suggested that, compared to most existing *in situ* hydrogels, PLGA and Mg@PEG-PLGA hydrogels displayed higher elastic moduli (ranging from 20.1 ± 3.3 to 38.9 ± 0.7 MPa), providing the needed mechanical support after implantation 36, 37.

In summary, these findings suggest that Mg@PEG-PLGA hydrogels, which exhibit highly advantageous characteristics, can be utilized as porous scaffolds for osteoporotic bone regeneration through *in situ* minimally invasive injection.

### H_2_ generation and pore analysis of the Mg@PEG-PLGA hydrogels

**Figure 2:**
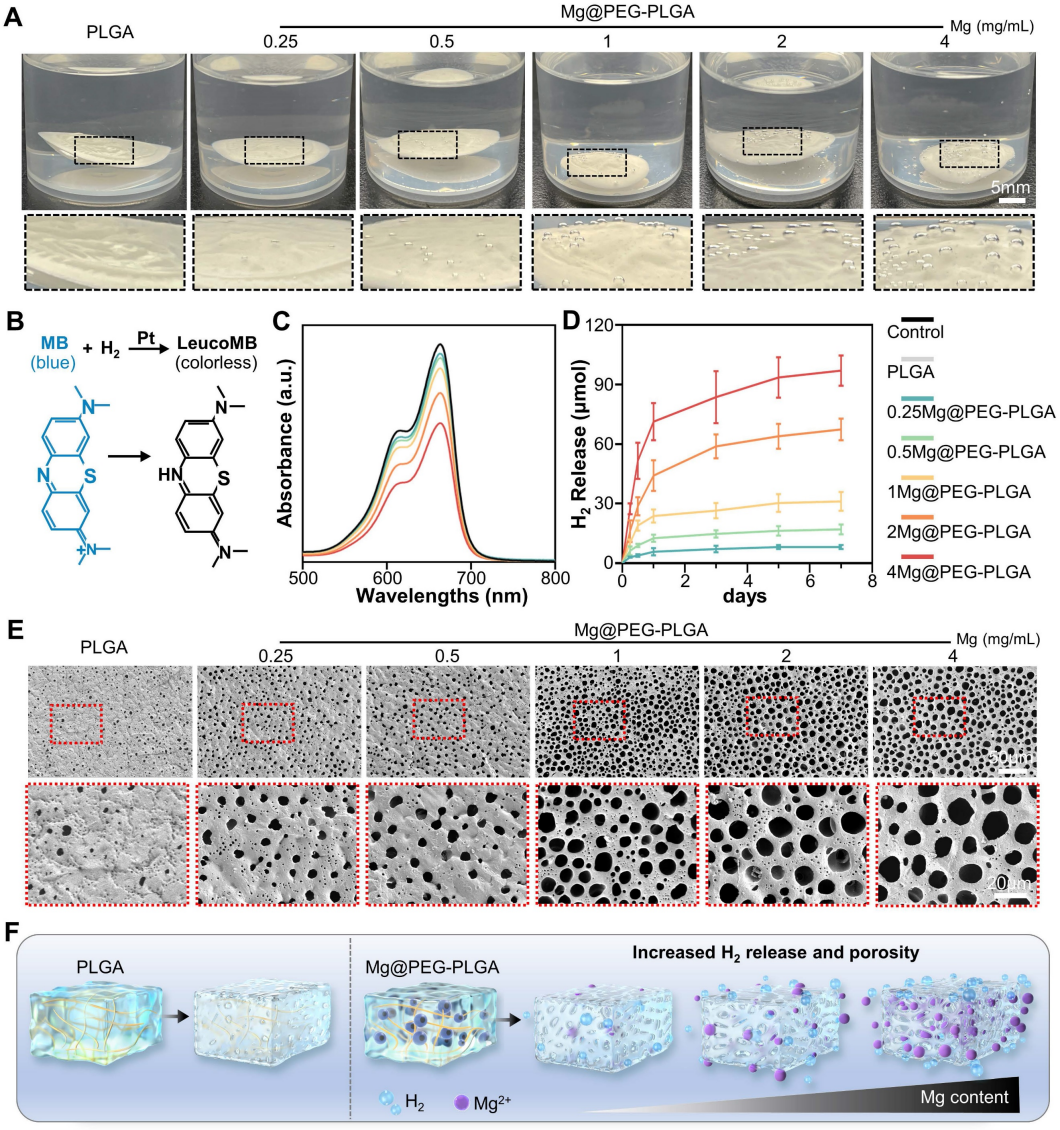
**H_2_ release and pore analysis of the Mg@PEG-PLGA hydrogels.** (A) Photograph showing H_2_ generation from the Mg@PEG-PLGA gels in PBS. (B) Schematic illustration of H_2_ generation determined by the MB-Pt probe solution. (C) Absorption spectra of the MB probe solution with or without the hydrogel added (6 h). (D) Time-dependent H_2_ generation measured by an MB probe from Mg@PEG-PLGA hydrogels. (E) SEM images of the solidified PLGA and Mg@PEG-PLGA gels. (F) Schematic illustration of the improved H_2_ generation ability of the Mg@PEG-PLGA gel with increased Mg content.

Molecular hydrogen, known for its high biosafety and excellent antioxidative ability, has been extensively investigated for clinical applications in various diseases 18. However, achieving sustainable and stable hydrogen (H_2_) delivery is challenging due to the short retention time and limited solubility of H_2_ in body fluids, which significantly limits its therapeutic efficacy 16, 22. Herein, the Mg@PEG-PLGA hydrogel offers a local H_2_ generation strategy for ROS scavenging through the following reaction: Mg + H_2_O → H_2_ + Mg(OH)_2_. As shown in **Figure 2A**, no significant gas bubbles were observed on the PLGA gel surface after immersion in PBS solution for 6 h. However, increased bubbles were observed in the Mg@PEG-PLGA groups with higher Mg contents. To evaluate H_2_ release from Mg@PEG-PLGA, an MB solution supplemented with Pt nanoparticles was used 23, 28, and the hydrogen content was determined based on the standard curve of MB absorbance at 664 nm** (Figure S8).** The reaction is shown in **Figure 2B**. As shown in **Figure 2C-D**, a significant initial burst release of H_2_ was detected in all the Mg@PEG-PLGA groups (0-12 h), likely because of the reaction between H_2_O and the Mg microparticles during the phase transition process. This initial burst release is crucial for alleviating ROS-mediated acute inflammation at the early stage of tissue damage 28, 38. Subsequently, the H_2_ release rate in the Mg@PEG-PLGA groups decreased, and the H_2_ release content increased with increasing Mg content: 0.25Mg@PEG-PLGA (8.24 ± 1.10 μmol) < 0.5Mg@PEG-PLGA (17.12 ± 2.16 μmol) < 1Mg@PEG-PLGA (32.54 ± 3.83 μmol) < 2Mg@PEG-PLGA (57.53 ± 5.40 μmol) < 4Mg@PEG-PLGA (71.32 ± 8.15 μmol). This result suggested the feasibility of adjusting H_2_ release by regulating the content of the incorporated Mg microspheres. Notably, 2Mg@PEG-PLGA and 4Mg@PEG-PLGA showed a sustained H_2_ release profile (up to 7 days), covering the inflammation and angiogenesis periods of bone regeneration 39. Porosity is an important characteristic of bone substitute materials and can affect matter transport, cell adhesion, vascularization, and bone growth 40, 41. It is generally believed that a porosity greater than 70% is required for the repair of cancellous bone 42. SEM and automatic pore analysis results** (Figure 2E** and **Figure S9)** demonstrated increased pore diameter and porosity in the Mg@PEG-PLGA groups with higher Mg contents, indicating that Mg microparticles can serve as porogens to improve the pore structure of PLGA gels** (Figure 2F)**. Critically, the 2Mg@PEG-PLGA and 4Mg@PEG-PLGA hydrogels, which had ideal maximum pore diameters (> 20 μm) and porosities (> 80%), were considered more favorable for nutrient/oxygen diffusion, cell ingrowth, and tissue regeneration than the other gels.

In summary, we have demonstrated that Mg@PEG-PLGA gels, especially 2Mg@PEG-PLGA and 4Mg@PEG-PLGA, exhibit suitable and sustainable H_2_ release in PBS, which is beneficial for early-stage remodeling of the bone environment in osteoporotic bone defects. Moreover, due to the H_2_ generated during the phase transition process, porous 2Mg@PEG-PLGA and 4Mg@PEG-PLGA scaffolds with increased pore diameter and porosity were formed, providing a favorable platform for cell and tissue ingrowth after implantation.

### Degradation and cell compatibility of the Mg@PEG-PLGA hydrogels

Numerous studies have shown that Mg^2+^ can stimulate the proliferation and osteogenic differentiation of BMSCs, exerting a positive influence on *in vivo* bone regeneration 25, 27. Therefore, achieving suitable and sustainable release of Mg^2+^ ions from Mg-containing orthopedic implants is crucial. As shown in **Figure S10A**, at 7 days, the 4Mg@PEG-PLGA gels showed a noticeable initial burst release (over 250 μg/mL), which might impair osteogenesis 27. In contrast, the 0.5Mg@PEG-PLGA, 1Mg@PEG-PLGA, and 2Mg@PEG-PLGA gels exhibited stable and sustainable Mg^2+^ release. Importantly, osteogenesis-inducing Mg concentrations (approximately 50-200 μg/mL) were observed in the 1Mg@PEG-PLGA and 2Mg@PEG-PLGA groups after 7 and 14 days, respectively 27. Furthermore, it has been reported that due to its acidic degradation byproducts, PLGA can lead to acid buildup and local inflammation 33, 43. As shown in **Figure S10B**, a significant decrease in pH was observed in the PLGA and 0.25Mg@PEG-PLGA groups. However, the 0.5Mg@PEG-PLGA, 1Mg@PEG-PLGA, and 2Mg@PEG-PLGA groups exhibited relatively stable pH values throughout the 28-day immersion period (pH values: 6.90-7.48, 6.95-7.56, and 7.02-7.85, respectively). This result suggested that the appropriate incorporation of basic Mg-based materials can neutralize the acidic degradation products of PLGA, potentially increasing its biocompatibility and enabling broader applications.

In addition to the desired physical properties, high biocompatibility is imperative for the application of biodegradable hydrogels as bone repair materials 44. To assess the cytocompatibility of the PLGA and Mg@PEG-PLGA gels, mouse embryonic fibroblasts (MEFs) and RAW264.7 cells were cultured with different gels, and cell viability was quantitatively determined using a CCK-8 assay. As shown in **Figure S11**, the PLGA, 0.25Mg@PEG-PLGA, 0.5Mg@PEG-PLGA, 1Mg@PEG-PLGA, and 2Mg@PEG-PLGA gels displayed favorable cytocompatibility, while the cell viability in the 4Mg@PEG-PLGA group was significantly lower at 24 and 48 h than that in the control group. This observation, coupled with the results of the degradation test, suggested that this effect may be attributed to the harsh alkaline conditions and the excessive release of magnesium ions from the 4Mg@PEG-PLGA gel.

As mentioned earlier, the 2Mg@PEG-PLGA gel, which exhibits suitable H_2_ release, pore structure, and cytocompatibility, emerged as the preferred choice for subsequent bioactivity tests.

### Antioxidant properties of the 2Mg@PEG-PLGA hydrogel

Biomaterials incorporating various antioxidants, such as dopamine-modified oligonucleotides and manganese-containing bioceramics, have been extensively explored for their ability to promote osteoporotic bone regeneration by scavenging ROS 10, 45. However, current materials present challenges in clinical application due to their unstable bioactivity, potential cytotoxicity, and poor tissue penetration. Recently, hydrogen-mediated gas therapy has attracted increasing attention for ROS-related diseases because molecular hydrogen can exclusively quench detrimental reactive oxidants without disturbing the physical functions of other ROS 20, 28. Moreover, compared to conventional drugs and materials, hydrogen, which has a relatively small molecular mass, can quickly spread and penetrate cell membranes, exhibiting desirable biological effects in various acute and chronic inflammatory diseases 18, 38.

**Figure 3:**
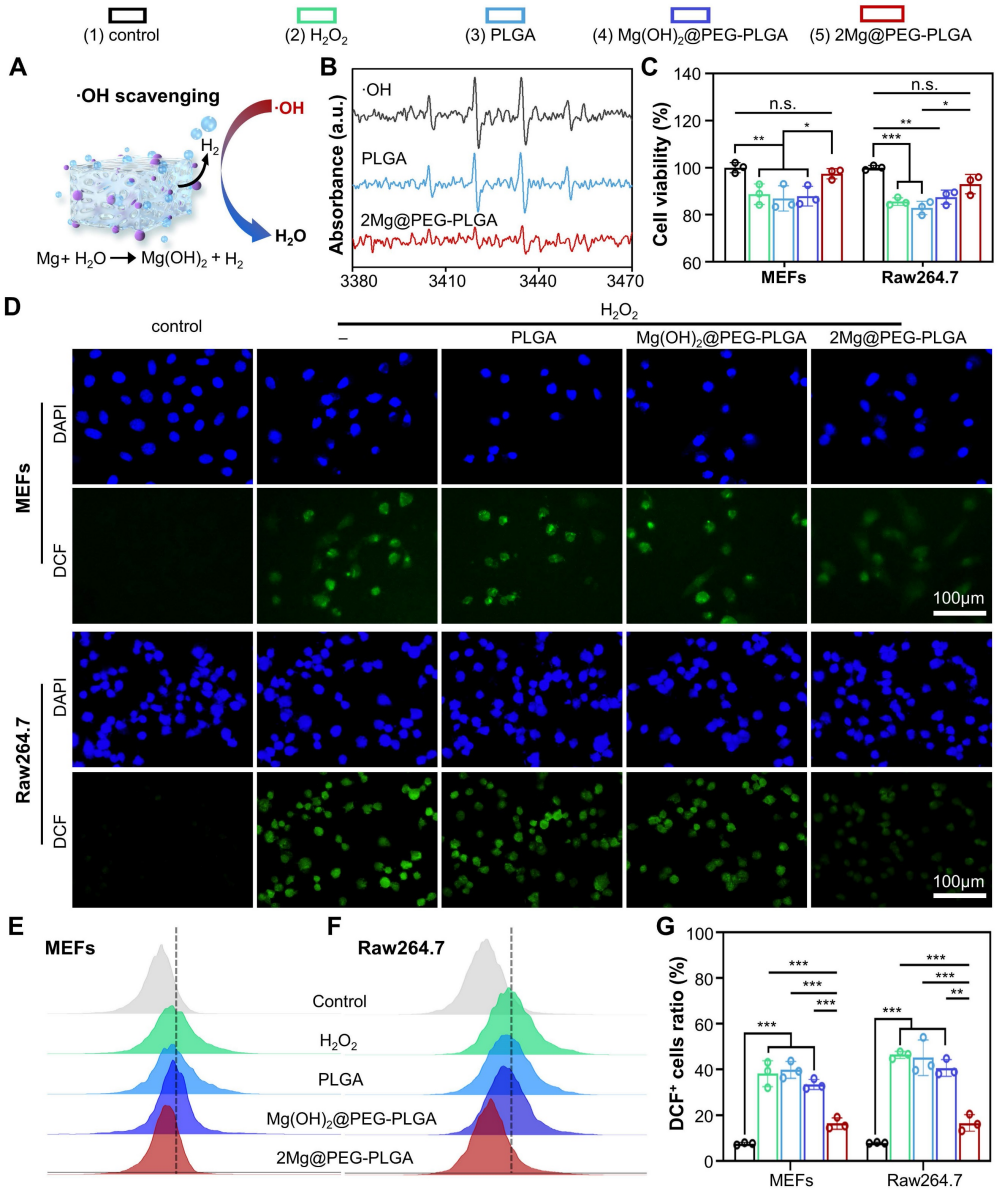
** ROS scavenging analysis of the 2Mg@PEG-PLGA hydrogel.** (A) Schematic illustration of ·OH reacting with H_2_ generated from the 2Mg@PEG-PLGA hydrogel. (B) The ·OH scavenging effect of 2Mg@PEG-PLGA detected by ESR. (C) Viability of H_2_O_2_-stimulated MEFs and RAW264.7 cells without or with hydrogel treatment. (D) ROS scavenging in MEFs and RAW264.7 cells after different treatments. (E-F) FCM results showing the intracellular ROS levels in MEFs and RAW264.7 cells after different treatments and (G) the corresponding quantitative analysis. The data are expressed as the mean ± SD (n=3). n.s. indicates no significance. *p < 0.05, **p < 0.01 and ***p < 0.001.

As depicted in **Figure 3A-B**, compared with that of other gels, the hydrogen generation ability of 2Mg@PEG-PLGA significantly decreased the characteristic peak intensities of BMPO/•OH, indicating that this material has excellent hydroxyl radical (•OH) scavenging ability. The ability of 2Mg@PEG-PLGA to scavenge excessive ROS in cells was assessed using MEFs and RAW264.7 cells. To control the influence of Mg^2+^ ions, the Mg(OH)_2_@PEG-PLGA group (loaded with the same Mg content as 2Mg@PEG-PLGA) was used in subsequent experiments. According to the CCK-8 results (**Figure 3C**), the antioxidant protection effect of the 2Mg@PEG-PLGA hydrogel was significantly greater than that of the other groups. Additionally, to determine the extent of cellular ROS scavenging, 2',7'-dichlorofluorescin diacetate (DCFH-DA), which strongly emits green fluorescence (DCF) upon oxidation by cellular esterases, was used as a ROS indicator. As shown in** Figure 3D-G**, strong green DCF fluorescence signals were observed in H_2_O_2_-stimulated MEFs and RAW264.7 cells, indicating abundant ROS generation. No significant change in ROS levels was observed in the PLGA or Mg(OH)_2_@PEG-PLGA gel groups compared with those in the H_2_O_2_ group. In contrast, the 2Mg@PEG-PLGA gel significantly reduced the intracellular ROS levels in H_2_O_2_-treated MEFs and RAW264.7 cells** (Figure 3G)**. Overall, these results indicate that the 2Mg@PEG-PLGA gel can effectively protect different cells against ROS-mediated damage by annihilating detrimental hydroxyl radicals, showing great potential for ROS scavenging in osteoporosis.

### *In vitro* immunomodulatory effect of the 2Mg@PEG-PLGA hydrogel

**Figure 4:**
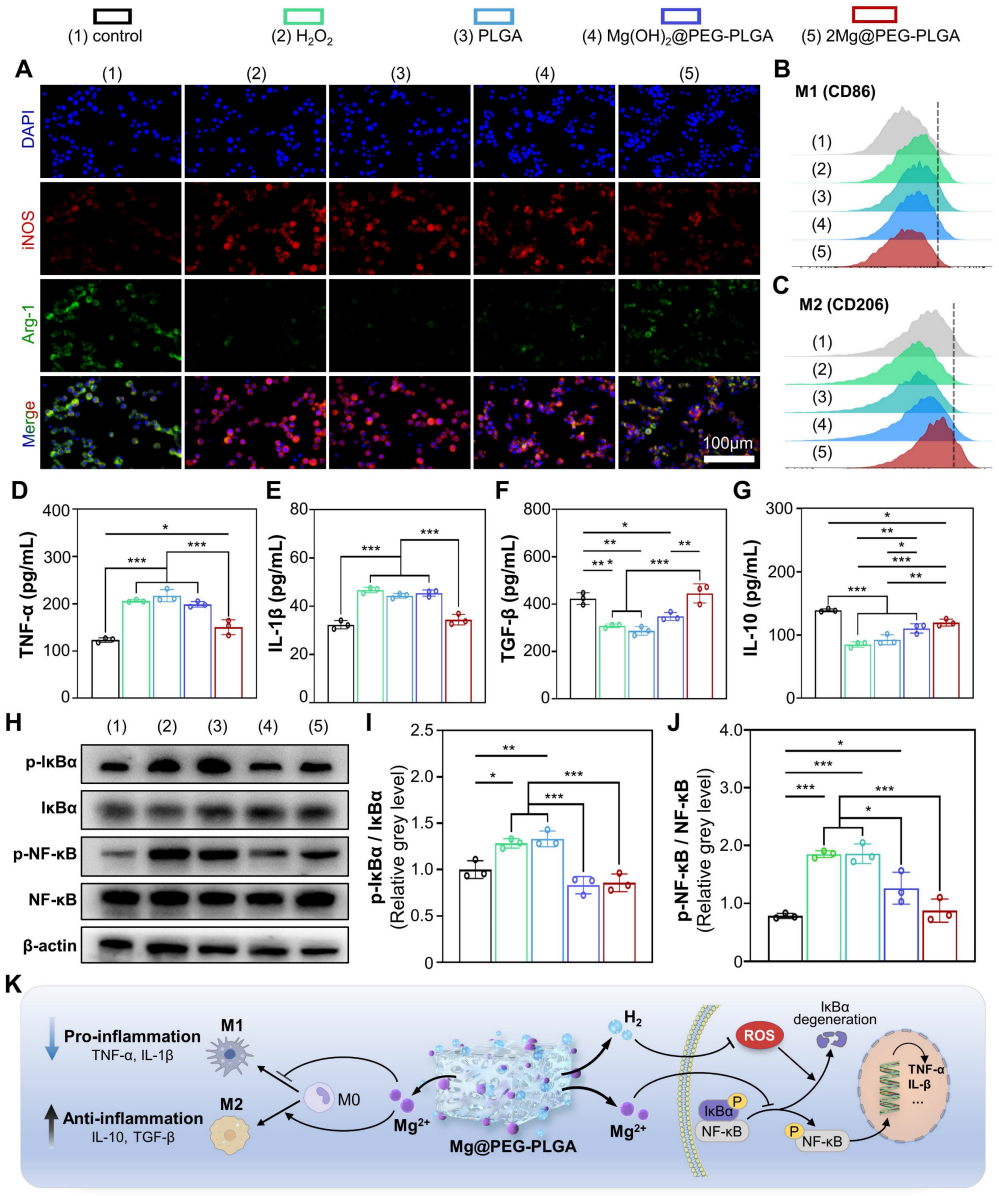
** Immunomodulatory properties of the 2Mg@PEG-PLGA hydrogel.** (A) Immunofluorescence images of iNOS, Arg-1 and DAPI staining of macrophages in the different groups. FCM results of (B) M1 (CD86+) and (C) M2 (CD206+) macrophages. (D-G) Secretion levels of TNF-α, IL-1β, TGF-β and IL-10 in macrophage suspensions. (H) Representative Western blot images of p-IκBα, IκBα, p-NF-κB, NF-κB and β-actin in the indicated groups. Quantitative analyses of the (I) p-IκBα/IκBα and (J) p-NF-κB/NF-κB ratios. (K) Schematic illustration of the immunomodulatory mechanism of the 2Mg@PEG-PLGA gel. The data are expressed as the mean ± SD (n=3). *p < 0.05, **p < 0.01 and ***p < 0.001.

Macrophages play a crucial role in the initiation and maintenance phases of tissue repair, particularly in the regulation of phenotypic polarization 46. M1 macrophages are proinflammatory, while M2 macrophages alleviate inflammation and promote bone defect regeneration 46. As shown in** Figure 4A** and **Figure S12**, there was a significant increase in the amount of iNOS (a marker of M1 macrophages) and a decrease in the amount of Arg-1 (a marker of M2 macrophages) in the H_2_O_2_ group compared to the control group, indicating that M1 macrophage polarization was enhanced and M2 polarization was inhibited in response to H_2_O_2_. In contrast, the 2Mg@PEG-PLGA group exhibited a significant reduction in iNOS levels and an increase in Arg-1 levels compared to the H_2_O_2_ group. These results demonstrated that, compared with H_2_O_2_ alone, 2Mg@PEG-PLGA significantly reduced M1 macrophage polarization and induced M2 macrophage polarization. Similar results were observed via FCM analysis, in which M1 macrophages were marked by CD86 and M2 macrophages by CD206 **(Figure 4B-C** and **Figure S13)**. Furthermore, the levels of proinflammatory cytokines (TNF-α and IL-1β) and anti-inflammatory cytokines (TGF-β and IL-10), which are typical biomarkers, were examined to evaluate the immunomodulatory effect of 2Mg@PEG-PLGA **(Figure 4D-G)**. The results showed that the secretion of the cytokines TNF-α and IL-1β was significantly lower in the 2Mg@PEG-PLGA group than in the H_2_O_2_ group, while the secretion of TGF-β and IL-10 was significantly greater, indicating the superior anti-inflammatory activity of the 2Mg@PEG-PLGA gel.

The NF-κB signaling pathway, which involves redox-sensitive transcription factors, regulates inflammation, osteoblastic differentiation, and cell apoptosis 47. Many studies have shown that excessive ROS lead to abnormal activation of the NF-κB signaling pathway, and similar results were observed in the H_2_O_2_ group, in which the phosphorylation ratio of IκBα and NF-κB proteins was significantly increased **(Figure 4H-J)** 47. However, compared to that in the H_2_O_2_ group, the phosphorylation ratio of IκBα to NF-κB was significantly lower in the 2Mg@PEG-PLGA group, indicating the inhibition of the IκBα/NF-κB signaling pathway. Notably, the NF-κB pathway was also downregulated in the Mg(OH)_2_@PEG-PLGA group. These results suggest that, in addition to the alleviation of oxidative stress by H_2_, the underlying mechanism of the downregulation of the NF-κB signaling pathway may be related to the Mg^2+^ ions in the 2Mg@PEG-PLGA group, which can inhibit the degradation of IκB and the production of free NF-κB 25, 27.

As mentioned above, the 2Mg@PEG-PLGA hydrogel exerts a significant anti-inflammatory effect by regulating macrophage polarization and inhibiting the IκBα/NF-κB pathway** (Figure 4K)**, which may provide a beneficial immune microenvironment for osteogenesis *in vivo*.

### *In vitro* antiosteoclastogenic and pro-osteogenic properties of the 2Mg@PEG-PLGA hydrogel

**Figure 5:**
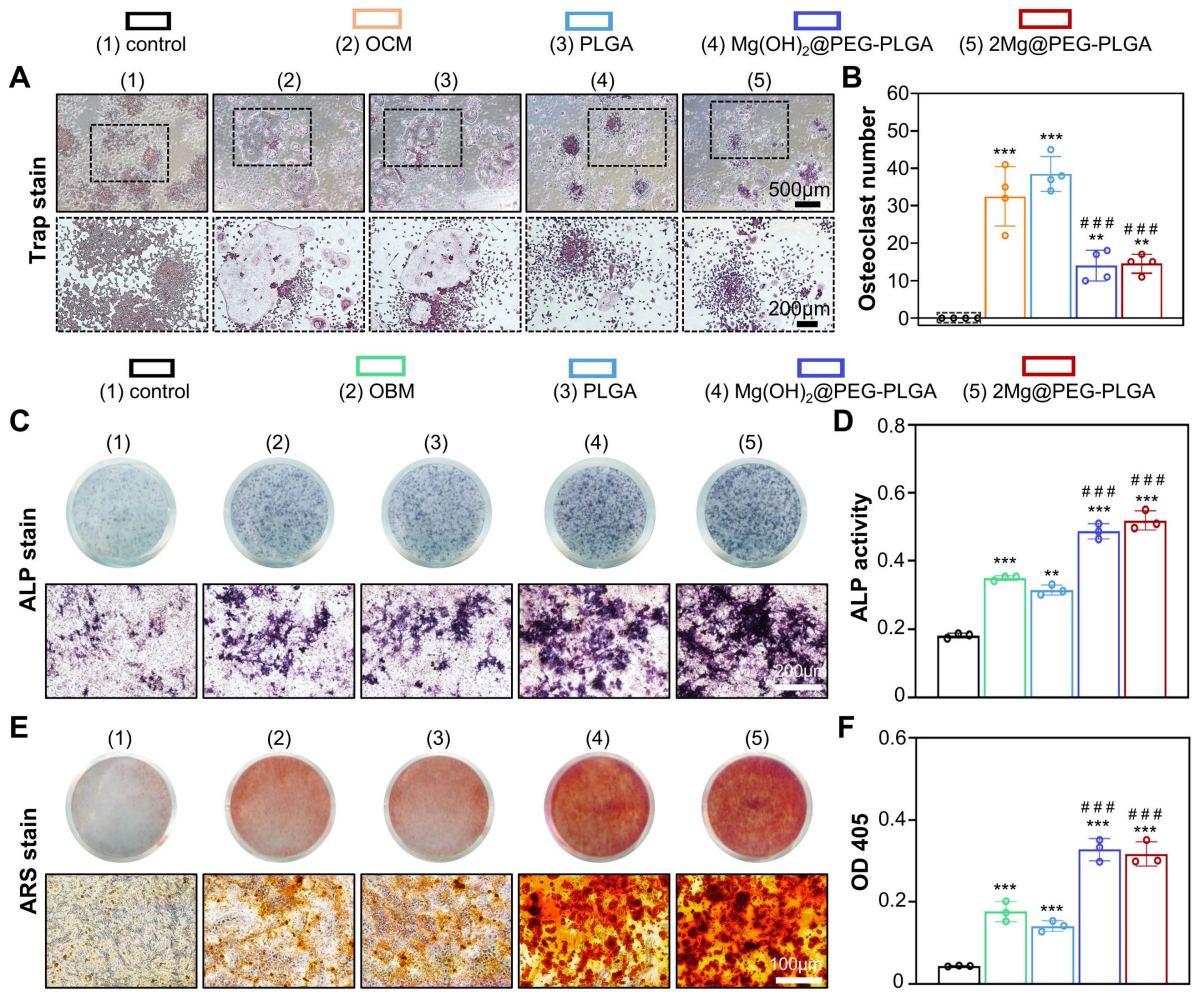
***In vitro* antiosteoclastic and pro-osteoblastic properties of the 2Mg@PEG-PLGA hydrogel.** (A) TRAP staining of RAW264.7 cells cultured in OCM supplemented with or without different hydrogels for 7 days and (B) corresponding quantitative analysis of TRAP+ cells per well. (C) ALP staining and (D) ALP activity quantitative analysis of MEFs after culture with OBM with or without supplementation with different hydrogels for 7 days. (E-F) ARS staining and corresponding quantitative analysis of MEFs after culture with OBM supplemented with or without different hydrogels for 14 days. The data are expressed as the mean ± SD. **p < 0.01 and ***p < 0.001, compared with the control group; ^###^*p*<0.001, compared with the OCM (Figure 5B) or OBM (Figure 5D and Figure 5F) group.

In osteoporosis patients, the balance between bone formation and resorption is disrupted due to excessive osteoclastogenesis and the attenuated osteogenic differentiation of BMSCs, which presents challenges in treating osteoporotic bone defects 2, 8. In this context, RAW264.7 cells were used to investigate the effect of the 2Mg@PEG-PLGA gel on osteoclastogenesis. After osteoclastic induction, there was no significant difference in osteoclastic enzymatic activity (analyzed by TRAP staining) between the OCM and PLGA groups **(Figure 5A-B)**. However, a decreased number of TRAP+ multinucleated osteoclasts was observed in the Mg(OH)_2_@PEG-PLGA and 2Mg@PEG-PLGA groups. The inhibition of osteoclastic differentiation may be attributed to the release of Mg^2+^ ions, which are known to inhibit osteoclastogenesis both* in vitro* and *in vivo* 25. Furthermore, MEFs with multipotent differentiation potential were used to evaluate the osteoinductive ability of the 2Mg@PEG-PLGA gel 48, 49. As shown in** Figure 5C-D**, compared with the OBM group, the 2Mg@PEG-PLGA group exhibited substantially increased ALP protein levels and activity. Additionally, according to the ARS staining analysis** (Figure 5E-F)**, the biomineralization of MEFs significantly increased after treatment with the 2Mg@PEG-PLGA gel.

In summary, the 2Mg@PEG-PLGA gel, which exhibits excellent antiosteoclastogenic and pro-osteogenic properties* in vitro*, is expected to accelerate osteoporosis-related bone regeneration *in vivo* by addressing the imbalance between osteoblastic bone formation and osteoclastic bone resorption.

### Ultrasound-guided minimally invasive implantation of the Mg@PEG-PLGA hydrogel

Bone implants would benefit from minimally invasive attributes to reduce surgical trauma and therefore time to heal 50. As shown in **Figure S14**, a rabbit model of femoral condylar defects (5 mm in diameter, 3 mm in depth) was established to evaluate the potential of ultrasound-guided minimally invasive implantation of the Mg@PEG-PLGA hydrogel. After locating the femoral condylar defect, a needle (16 G) was slowly inserted into the defect site under continuous ultrasound guidance (**Figure S14C**). Approximately 25 μL of the hydrogel was successfully injected to fill the bone defect. Following a 5-min local infusion of 0.9% saline, a solidified Mg@PEG-PLGA hydrogel with increased echo intensity was observed. These results indicate the feasibility of using Mg@PEG-PLGA hydrogels combined with ultrasound guidance for the minimally invasive treatment of bone defects.

### *In vivo* osteoporotic bone defect repair efficacy of the 2Mg@PEG-PLGA hydrogel

**Figure 6:**
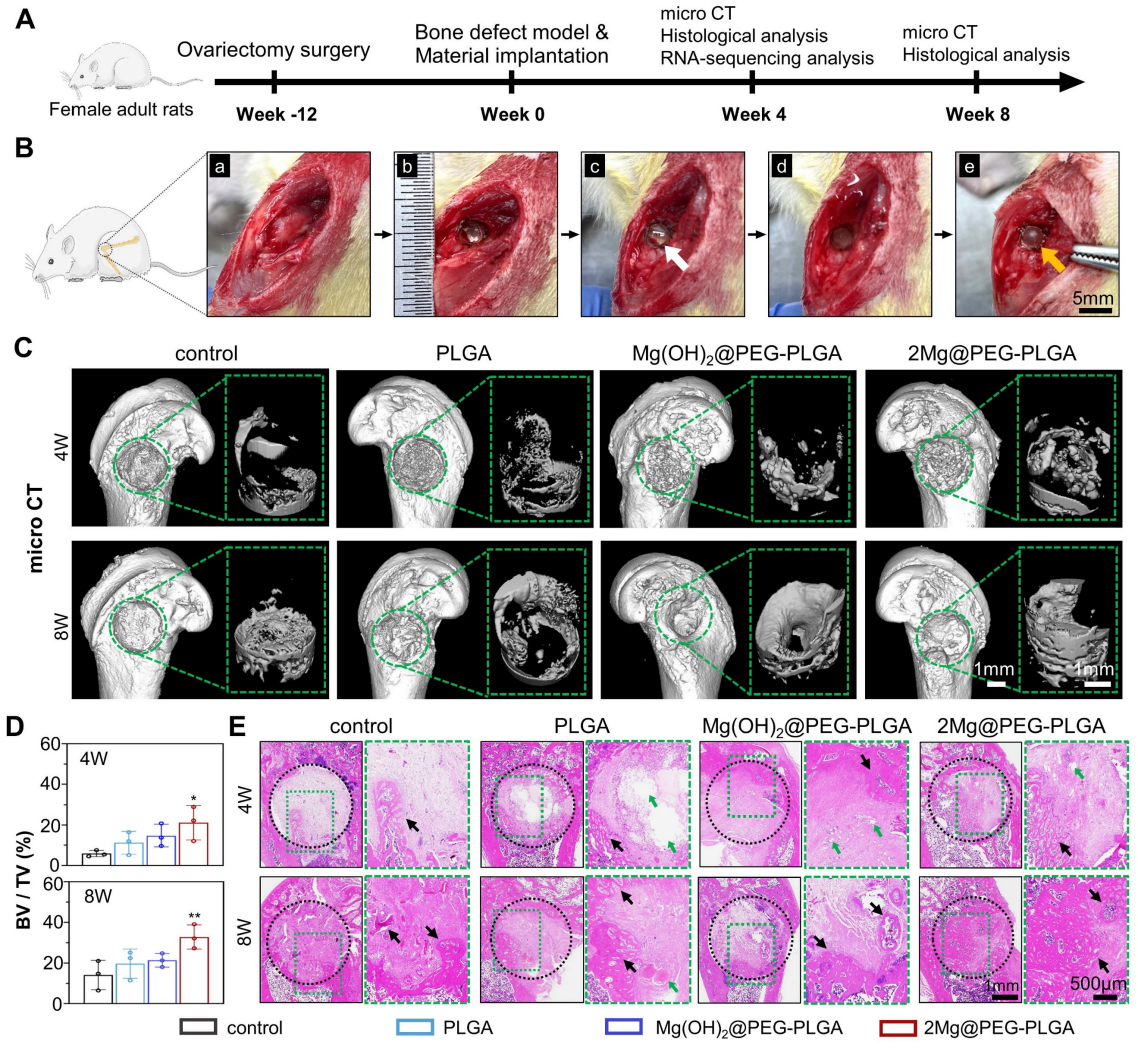
** Osteoporotic bone defect repair efficacy of the 2Mg@PEG-PLGA hydrogel.** (A) Schematic timeline of the *in vivo* study. (B) The surgical process of *in situ* implantation of the 2Mg@PEG-PLGA hydrogel in osteoporotic bone defects. (a-b) The construction of bone defects (3 mm in diameter × 3 mm in depth) on the lateral epicondyle of the femur. (c-e) The implanted 2Mg@PEG-PLGA hydrogel was solidified after immersion in saline for 5 min. The white and red arrows indicate gelatinous and solidified 2Mg@PEG-PLGA gels, respectively. (C) Micro-CT 3D-reconstructed images of the distal femur of rats and the newly formed bone within the bone defect at 4 and 8 weeks. The green circle marks the bone defects. (D) BV/TV analysis of the newly formed bone within the bone defect via micro-CT. (E) HE staining of rat femurs from different groups at 4 and 8 weeks. The black circle marks the bone defects. The green arrows indicate residual materials. The black arrows indicate the newly formed bone. The data are expressed as the mean ± SD (n = 3). *p < 0.05 and **p < 0.01.

The osteoporotic bone defect repair capability of 2Mg@PEG-PLGA was evaluated using an established osteoporotic femur defect model in OVX rats** (Figure 6A)**. Rats underwent either OVX or sham surgery. Three months after bilateral OVX, the micro-CT images of the vertebral body revealed a more disordered trabecular microstructure and enlarged cavities in the OVX rats than in the sham rats** (Figure S15)**. Quantitative analysis of the bone parameters demonstrated decreased BV/TV, BMD, and Tb.Sp in the OVX rats. H&E and Masson staining further revealed extensive bone loss induced by the OVX procedure in the rat femur and vertebral body** (Figure S16)**, confirming the establishment of osteoporosis in female OVX rats. Subsequently, a burr hole defect was created in the rat femur, which was treated with the *in situ-*formed composite gel, as illustrated in **Figure 6B**. The implant site of the rat femur was exposed 3 days after Mg@PEG-PLGA hydrogel implantation (**Figure S17**). There was no observable hydrogel leakage into the surrounding tissue, and the solidified hydrogel was tightly adherent to the surrounding bone tissue, indicating good adhesion of the Mg@PEG-PLGA hydrogel to the bone defect site after *in vivo* implantation.

At weeks 4 and 8, the regeneration of osteoporotic bone defects was evaluated via micro-CT and histological analysis. Representative 3D images of new bone formation in the femur defects clearly depicted differences among the four groups** (Figure 6C)**. Unlike the small amount of new bone tissue that formed at the margin of the defects in the control, PLGA, and Mg(OH)_2_@PEG-PLGA groups, the defect in the 2Mg@PEG- PLGA group was almost filled with new bone at 8 weeks postoperation. According to the results of quantitative micro-CT analysis, the 2Mg@PEG-PLGA group showed significantly greater new bone quantity at both 4 and 8 weeks postoperation than did the control group** (Figure 6D)**. HE staining revealed that the defect region in the control group was filled with fibrous tissue at week 8, suggesting that such a bone defect could not self-repair under osteoporotic conditions** (Figure 6E)**. In the PLGA and Mg(OH)_2_@PEG-PLGA groups, only a small quantity of bone had formed at the defect margins at 4 and 8 weeks postoperation. Notably, at 4 weeks postoperation, significantly increased bone regeneration was observed in the 2Mg@PEG-PLGA group, in which almost half of each defect was filled with newly formed bone tissue. Accompanied by the complete degradation of the 2Mg@PEG-PLGA gel at 8 weeks, the defect region was filled with remodeled bone tissue with numerous marrow spaces, demonstrating the superior efficacy of 2Mg@PEG-PLGA hydrogels in repairing osteoporotic bone defects *in vivo*.

**Figure 7:**
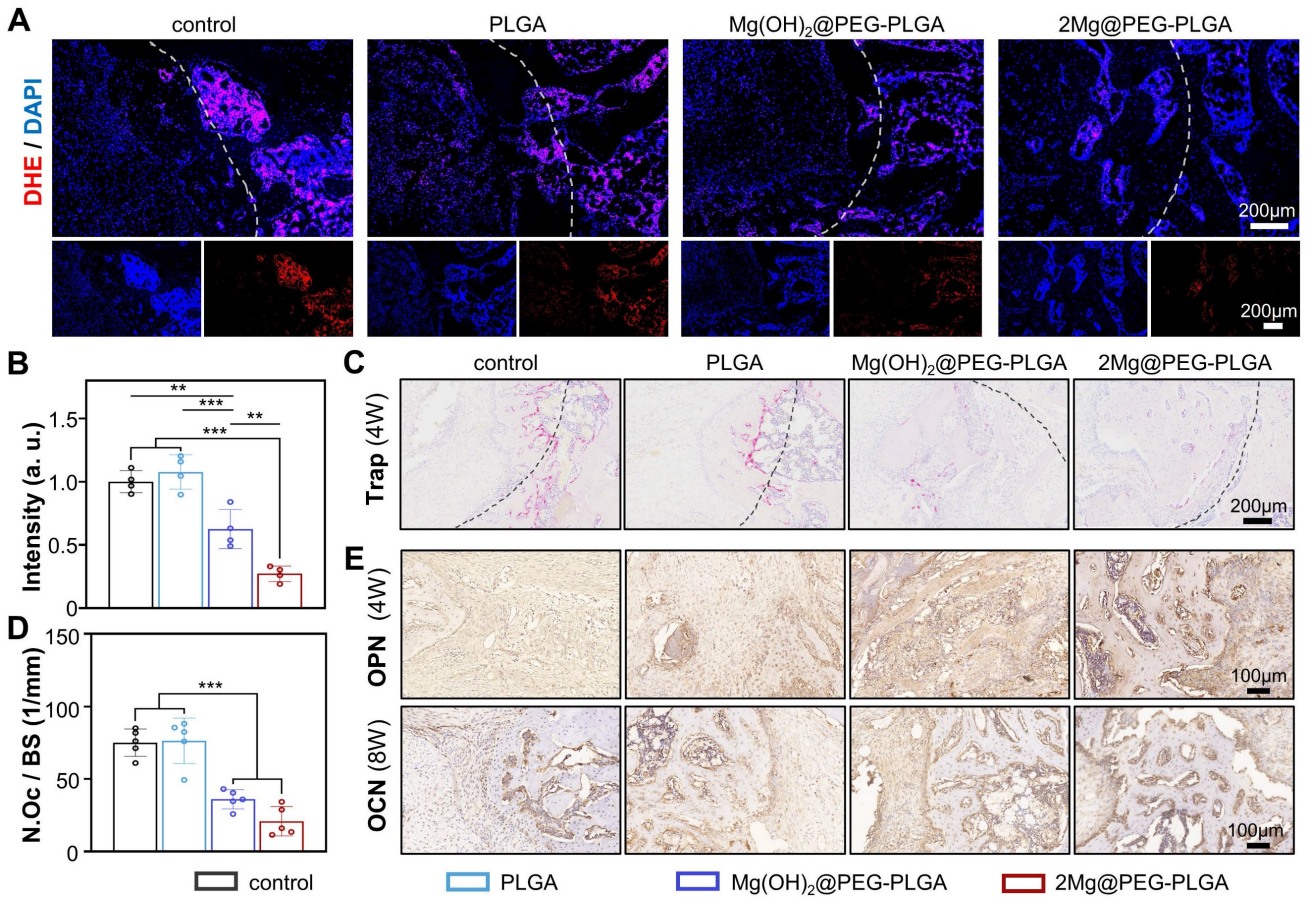
** The ability of the 2Mg@PEG-PLGA hydrogel to scavenge ROS, inhibit osteoclastogenesis, and promote osteogenesis *in vivo*.** (A) Representative images and (B) corresponding quantification of DHE staining at the bone defect site (week 4). The white dotted lines mark the bone defects. (C) TRAP staining and (D) corresponding quantification of the number of TRAP+ osteoclasts at the bone defect sites (week 4). The black dotted lines mark the bone defects. (E) Images of immunohistochemical staining for OPN and OCN in bone defects. The data are expressed as the mean ± SD; **p < 0.01 and ***p < 0.001.

In addition, the defect areas were analyzed by DHE, TRAP, and IHC staining to investigate the bone regeneration process at the cellular and molecular levels. To assess the *in vivo* ROS scavenging ability of 2Mg@PEG-PLGA, the DHE probe was applied as previously reported 51. Consistent with the findings of previous publications, ROS levels were highly elevated after the OVX procedure, suggesting that a harsh environment was not conducive to osteoporotic bone regeneration. As shown in** Figure 7A-B**, the 2Mg@PEG-PLGA gel dramatically reversed local ROS production within the implantation area. Moreover, TRAP staining confirmed the antiosteoclastic property of the 2Mg@PEG-PLGA gel *in vivo* **(Figure 7C-D)**. Additionally, the expression of osteogenic markers (OPN and OCN) in the defect area was evaluated via IHC. As shown in** Figure 7E**, the highest OPN and OCN expression levels were observed in the 2Mg@PEG-PLGA group, indicating increased osteogenesis and new bone regeneration. Interestingly, decreased ROS levels and osteoclast numbers were also observed in the Mg(OH)_2_@PEG-PLGA group. It has been reported that under osteoporotic conditions, overactivated osteoclasts are one of the main sources of excessive ROS 14, 52. Taken together, these findings suggest that the Mg^2+^ ions released from Mg-based implants may scavenge ROS in osteoporosis via the inhibition of osteoclastogenesis.

**Figure 8:**
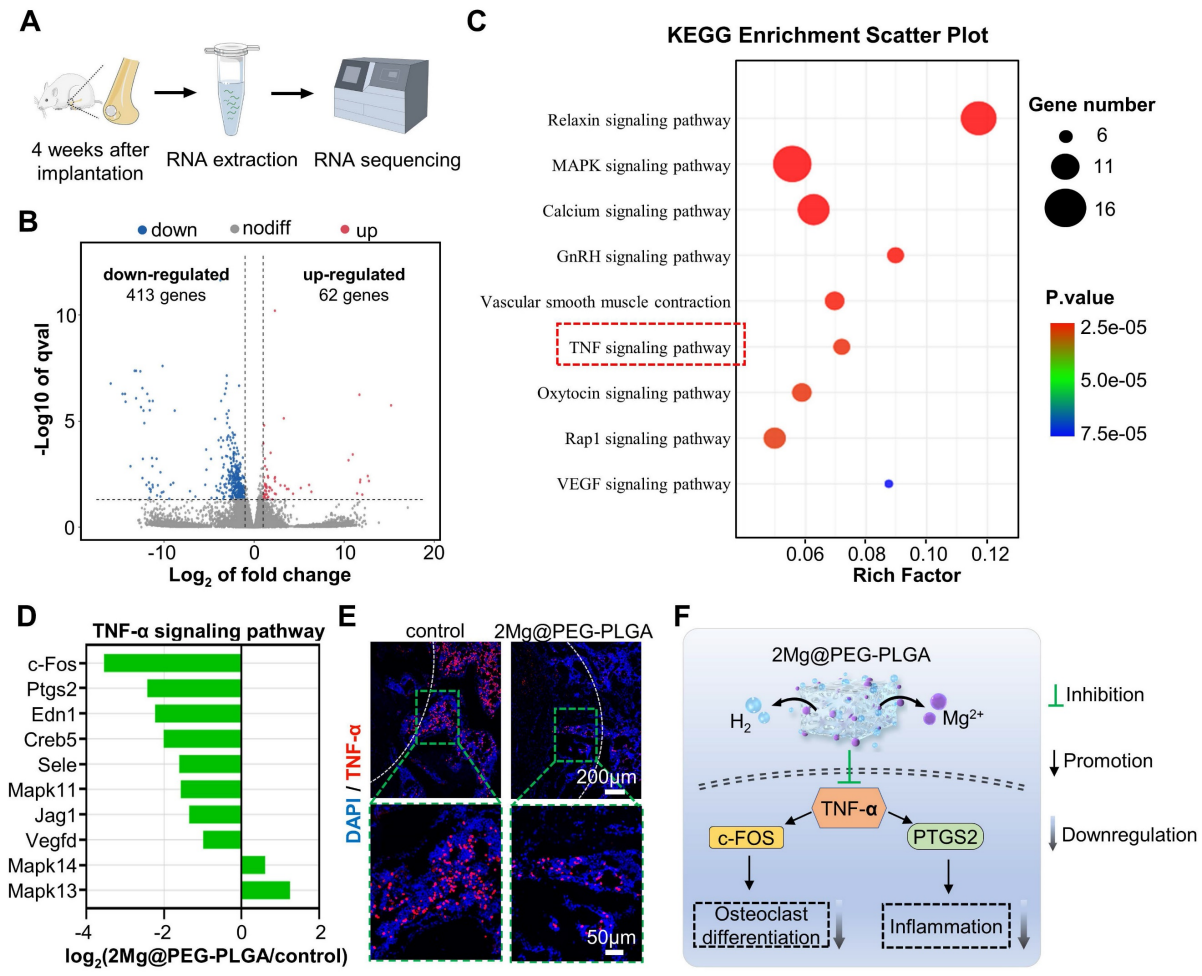
** Transcriptome high-throughput sequencing was used to study the effect of the 2Mg@PEG-PLGA gel on osteoporotic bone defect repair.** (A) Schematic diagram of the RNA sequence analysis of extracted tissues within femoral bone defects from the control and 2Mg@PEG-PLGA groups at 4 weeks. (B) Volcano plot of genes that were differentially expressed between the control and 2Mg@PEG-PLGA groups. (C) Representative KEGG pathways associated with genes that were significantly differentially expressed between the control and 2Mg@PEG-PLGA groups. (D) Differentially expressed genes involved in the TNF-α signaling pathway. (E) IF images of TNF-α protein expression in femoral bone defects at 4 weeks. The white dotted lines mark the bone defects. Red fluorescence: TNF-α; blue fluorescence: DAPI. (F) Schematic illustration of the mechanism by which 2Mg@PEG-PLGA downregulates osteoclastogenesis and inflammation by inhibiting the TNF-α signaling pathway.

To further investigate the intrinsic mechanism of 2Mg@PEG-PLGA-mediated osteoporotic bone regeneration, we conducted high-throughput RNA-seq of bone tissues from the control and 2Mg@PEG-PLGA groups at 4 weeks** (Figure 8A)**. The heatmap (**Figure S18**) clearly shows a greater number of downregulated genes (in blue) than upregulated genes (in orange). A volcano plot revealed that the expression of 62 genes was significantly upregulated and that of 413 genes was markedly downregulated in the 2Mg@PEG-PLGA group compared with the control group** (Figure 8B)**. GO enrichment analysis indicated that these genes significantly participated in several biological processes, including cell adhesion, negative regulation of monocyte chemotaxis, and positive regulation of the cell migration process **(Figure S19)**. To better understand the biological functions of the differentially expressed genes (DEGs) in the 2Mg@PEG-PLGA gel, KEGG pathway enrichment was performed, and the results showed that the MAPK signaling pathway, calcium signaling pathway, GnRH signaling pathway, and TNF-α signaling pathway were highly involved in the therapeutic process of 2Mg@PEG-PLGA** (Figure 8C)**. Notably, the TNF-α signaling pathway, which plays a crucial role in inflammation and osteoclastic differentiation, was significantly inhibited in the 2Mg@PEG-PLGA group** (Figure 8D)** 53. To verify this result, we evaluated the level of TNF-α in the bone defect area. As expected, the TNF-α level in the 2Mg@PEG-PLGA group was obviously lower than that in the control group **(Figure 8E)**, which is consistent with the *in vitro* results mentioned above. Accordingly, we concluded that the 2Mg@PEG-PLGA gel can reduce inflammation and osteoclastogenesis *in vivo* by inhibiting the TNF-α signaling pathway, improving the osteoporotic bone regeneration microenvironment **(Figure 8F)**.

### *In vivo* biocompatibility analysis of the 2Mg@PEG-PLGA hydrogel

As reported by other researchers and our group, PLGA/NMP hydrogels are biodegradable and biocompatible and are widely used as nano/micro materials and drug carriers 31, 32. Moreover, the clinical product Eligard has been commercialized and applied for the treatment of advanced prostate cancer^44^. Herein, the* in vivo* biocompatibility of 2Mg@PEG-PLGA was investigated via histological and serological analysis of treated rats. First, the major organs of the experimental rats, including the heart, liver, spleen, lung, kidneys, and brain, were collected for pathological analysis via H&E staining** (Figure S20)**, and no obvious histological variation was observed in the 2Mg@PEG-PLGA group. Second, the serum ALT, AST, BUN, and CREA levels were within the normal reference ranges, indicating that liver and kidney function in the 2Mg@PEG-PLGA group was normal during the first 8 weeks after implantation** (Table S1)**. In addition, the serum Mg^2+^ concentration in the 2Mg@PEG-PLGA group was within the normal range during the observation period** (Table S1)**. These preliminary results showed that the 2Mg@PEG-PLGA gel can be considered a potentially nontoxic and biocompatible implant for effectively accelerating osteoporotic bone regeneration.

## Conclusion

This study encompasses a comprehensive evaluation, including* in vitro* and* in vivo* assessments, that provides insights into the potential mechanisms underlying the observed therapeutic effects. The developed 2Mg@PEG-PLGA gel not only addresses the challenges associated with osteoporotic bone defects but also offers the advantages of injectability, controlled H_2_ release, and modulation of the bone microenvironment. This study contributes to the field of bone regeneration by introducing a novel approach that combines the benefits of H_2_ therapy and Mg^2+^ ions for treating osteoporotic bone defects. These *in vitro* and *in vivo* results support the notion that the 2Mg@PEG-PLGA gel effectively promotes bone regeneration by modulating inflammation, osteoclastogenesis, and the TNF-α signaling pathway.

Importantly, this study's success introduces opportunities for further research and potential clinical applications. These promising results encourage the exploration of similar strategies in other models or the optimization of gel formulations for broader applicability. Additionally, long-term studies assessing the sustained effects and safety profiles of the 2Mg@PEG-PLGA gel would be valuable for future clinical translation.

Overall, this study demonstrated the potential of the developed 2Mg@PEG-PLGA gel as an innovative and effective tool for treating osteoporotic bone defects, with a unique combination of injectability, controlled gas release, and immunomodulatory properties.

## Experimental section

### Materials

Magnesium microparticles (~50 μm) were procured from Shanghai Naiou Nanotechnology Co., China. Polyvinylpyrrolidone (PVP, MW = 40000) was obtained from Sigma‒Aldrich (USA), while polyethylene glycol (PEG, MW = 2000), ethyl acetate, and 1-methyl-2-pyrrolidinone (NMP) were obtained from Aladdin (Shanghai, China). Poly(lactic-co-glycolic acid) (PLGA, MW = 40000, 50:50) was obtained from Jinan Daigang Biomaterial Co., China. All additional chemical reagents utilized in this study were of analytical purity and required no further purification.

### Preparation of Mg@PEG microparticles

To fabricate Mg@PEG microspheres featuring an asymmetrical PEG coating, an embedding method was employed 23, 28. Briefly, Mg microparticles were thoroughly washed with acetone to eliminate any excess MgO layer. Subsequently, a fine layer of polyvinylpyrrolidone (PVP) was delicately sprayed onto a glass plate to provide a foundation for the Mg microparticles. The next step involved the uniform distribution of Mg microparticles on the PVP-coated surface. Next, 2 wt% PEG solution, dissolved in ethyl acetate, was carefully added dropwise to the Mg microparticles. Upon completion of the deposition, the Mg@PEG microspheres were carefully collected by scratching them off the glass plate.

### Preparation of PLGA and Mg@PEG-PLGA hydrogels

The PLGA hydrogel was prepared following previously reported methods 31-33. In brief, PLGA particles were introduced into an NMP solution at a fixed mass-to-volume ratio, and the mixture was agitated at 37°C until the PLGA had fully dissolved. Subsequently, Mg@PEG microparticles were incorporated into the PLGA hydrogel at various mass ratios through mechanical stirring, resulting in the formation of Mg@PEG-PLGA hydrogels **(Table 1)**. To determine the potential impact of Mg^2+^ ions, a control hydrogel, Mg(OH)_2_@PEG-PLGA, loaded with a Mg content comparable to that of Mg@PEG-PLGA was prepared following the same procedure described above.

For biological analysis, all materials were sterilized by ultraviolet (UV) irradiation or sterilized by filtration 54, 55. Briefly, PLGA and synthesized Mg@PEG microparticles were exposed to ultraviolet (UV) irradiation for 30 min for sterilization. NMP solutions were filter sterilized with 0.22 μm syringe filters (Millex). Then, the Mg@PEG-PLGA hydrogel was prepared under sterile conditions.

### Characterization

The morphologies of the Mg and Mg@PEG particles were assessed through scanning electron microscopy (SEM) using a Zeiss Merlin Compact instrument from Germany after gold coating. Elemental mapping via energy dispersive spectroscopy (EDS) was conducted with the same parameters employed for SEM. Thermogravimetric analysis (TGA; TGA5500) was performed to determine the amount of Mg incorporated into the Mg@PEG microspheres. The injectability, liquid‒solid phase transition, and shape adaptability of the Mg@PEG-PLGA gels were documented through digital imaging. To evaluate the adhesion of the Mg@PEG-PLGA hydrogel to the bone defect site, approximately 20 μL of the hydrogel was injected into the femoral condylar defect (3 mm for both diameter and depth) of each rat. After 5 min of irrigation with 0.9% saline, the change in the Mg@PEG-PLGA hydrogel was recorded during the rotation test. X-ray diffraction (XRD) experiments were carried out using a Rigaku Ultima IV instrument from Japan. X-ray photoelectron spectroscopy (XPS) was performed with a Thermo Scientific K-Alpha apparatus. The mechanical properties of the solidified PLGA and Mg@PEG-PLGA gels were evaluated using an INSTRON universal material testing machine from the USA, applying a test speed of 1 mm/min until the sample reached 60% deformation. Hydrogel samples with uniform shapes (5 mm in diameter, 6 mm high) were prepared and subjected to compressive testing, and the elastic modulus was calculated as the slope of the linear regions corresponding to 0-5% strain in the stress‒strain curve. Infrared spectra were acquired using a Fourier transform infrared (FTIR) spectrometer (Nicolet 6700).

### H_2_ release and pore analysis of the Mg@PEG-PLGA hydrogels

H_2_ release was evaluated using a methylene blue (MB) probe based on a previously reported method 23, 28. MB (with a characteristic absorption peak at 664 nm) can react with H_2_ in the presence of Pt nanoparticles to produce leucomethylene blue (leucoMB) according to the following equation:

MB (blue) + 2H^+^ + 2e^-^ → leucoMB (colorless)

Briefly, 1 mL of each Mg@PEG-PLGA gel was introduced into the MB-Pt probe solution (MB: 30 μM, Pt: 2 w/w%, 10 mL). At specified time intervals, the MB-Pt probe solution was replaced, and the change in absorbance of the MB-Pt probe solution at 664 nm was monitored using a UV‒Vis spectrophotometer (UV-3101PC Shimadzu spectrometer). Subsequently, H_2_ release was quantified based on the MB standard curve.

After gold coating, the morphological characteristics and elemental mapping of the solidified PLGA and Mg@PEG-PLGA gels were examined through SEM. An automatic pore analyzer (AutoPore 9500, USA) was used to determine the pore structure of the solidified gels. All analyses were conducted in triplicate to obtain robust and reliable results.

### Degradation of the Mg@PEG-PLGA hydrogels

Samples (1 mL) of PLGA and Mg@PEG-PLGA (loaded with varying Mg concentrations) were added to 10 mL of PBS at 37°C for 4 weeks, during which the solution was changed weekly 56, 57. At each specified time point, the concentration of Mg^2+^ in the PBS was quantified using inductively coupled plasma‒optical emission spectrometry (ICP‒OES) with a PerkinElmer Optima 8000 instrument from the USA. A pH meter (Smart Sensor, China) was used to monitor changes in the pH of the PBS.

### Cell culture

RAW264.7 cells were procured from Procell Life Science & Technology Co., China, while multipotent mouse embryonic fibroblasts (MEFs) were obtained from the Molecular Oncology Laboratory, Department of Surgery, University of Chicago Medical Center, USA. Both RAW264.7 cells and MEFs were cultured in α-modified Eagle's medium supplemented with 10% fetal bovine serum (Lonsera, Uruguay) and 1% penicillin‒streptomycin (Beyotime, China) at 37°C under 5% carbon dioxide (CO_2_).

For osteoclastogenesis assays, osteoclastic induction medium (OCM) was prepared by supplementing the culture medium with 25 ng/mL M-CSF (PeproTech, Inc.) and 50 ng/mL RANKL (PeproTech, Inc.). Osteoblastic induction medium (OBM) was generated from the culture medium by adding 5×10^-5^ mol/L ascorbic acid (Sigma, USA), 1×10^-2^ mol/L β-sodium glycerophosphate (Solarbio, China), and 1×10^-8^ mol/L dexamethasone (Sigma, USA).

### Cellular compatibility assays

To assess the cellular compatibility of the hydrogels, 50 μL of hydrogel was placed in 24-well plates, and RAW264.7 cells (5×10^3^) or MEFs (5×10^3^) were cultured on the hydrogels for 48 h. The control group consisted of a culture system without any experimental materials. At predetermined time points, Cell Counting Kit-8 (CCK-8) (Dojindo, Japan) reagent (10% in culture medium) was added to each well, and the cells were incubated at 37°C for an additional hour. Subsequently, the absorbance of the supernatant was measured using a microplate reader (Thermo Fisher Scientific, USA) at 450 nm.

### ROS scavenging activity of the Mg@PEG-PLGA hydrogels

The •OH scavenging activity of the 2Mg@PEG-PLGA hydrogel was evaluated using electron spin resonance (ESR) with a Bruker EMXplus instrument from Germany. Initially, •OH radicals were generated as the control group using a TiO_2_/UV system under 340 nm ultraviolet light. Subsequently, the 2Mg@PEG-PLGA hydrogel was introduced to the prepared mixture and incubated for 60 min, after which any remaining •OH radicals were captured using DMPO.

For comparative analysis, the Mg(OH)_2_@PEG-PLGA hydrogel was used to control the influence of Mg^2+^ ions in subsequent evaluations. The intracellular ROS scavenging ability of the 2Mg@PEG-PLGA hydrogel was determined using a DCFH-DA assay kit. RAW264.7 cells or MEFs were seeded in the lower chamber of a 6-well transwell plate at a density of 1 × 10^4^ cells per well. After 24 h, the cells in the lower chamber were exposed to 200 µM H_2_O_2_ for 12 h. Subsequently, the composite hydrogels were introduced into the upper chamber. After an additional 12 h, the cells were treated with 10 µM DCFH-DA for 30 min and stained with DAPI for 15 min. The intracellular ROS levels were then assessed using a fluorescence microscope (Olympus BX53F, Japan) and flow cytometry (FCM) analysis. Additionally, cell viability was assessed through a CCK-8 assay.

### Western blot analysis

RAW264.7 cells were seeded in 6-well plates (1 × 10^4^ cells per well) and subjected to the aforementioned treatments. At the designated time points, protein was extracted from the cells and quantified using a BCA protein assay kit (Beyotime, China). Equal amounts of protein were loaded, separated via SDS‒PAGE, and subsequently transferred to PVDF membranes (0.22 μm, Millipore, USA). The target proteins, including IκBα, p-IκBα, NF-κB, p-NF-κB (Abcam), and β-actin (Proteintech), were detected using specific antibodies. The stained bands were visualized through a chemiluminescence detection system, and the gray values were analyzed using ImageJ software.

### *In vitro* polarization of macrophages

RAW264.7 cells (5 × 10^3^) were seeded in the lower chambers of a 6-well transwell plate and exposed to H_2_O_2_ (200 μM) for 12 h. Subsequently, various hydrogels were placed in the upper chamber to investigate their effects on macrophage polarization. After a 3-day incubation, the cells and supernatant were collected through centrifugation. First, FCM analysis was conducted to examine the specific surface markers of the polarized macrophages. The cells were stained with anti-CD86 PE (Invitrogen, USA) and anti-CD206 APC (Invitrogen, USA) to identify the M1 (CD86+) and M2 (CD206+) phenotypes, respectively. Second, the collected supernatant was utilized to assess the cytokines secreted by M1 (TNF-α, IL-1β) and M2 (TGF-β, IL-10) macrophages through enzyme-linked immunosorbent assay (ELISA) kits (Shanghai Huyu Biotechnology Co., China). Furthermore, RAW264.7 cells were treated as described above with 4% polyformaldehyde for 10 min and stained with iNOS (M1) and Arg-1 (M2). The stained cells were then observed and photographed through a fluorescence microscope.

### *In vitro* osteoclastic differentiation

RAW264.7 cells (5×10^3^ per well) were seeded into the lower chambers of 24-well plates and cultured overnight. The culture medium was then replaced with osteoclast inductive medium (OCM), and different hydrogels were added to the upper chamber. The wells treated with culture medium or OCM were designated the control group and OCM group, respectively. After a 7-day induction period, tartrate-resistant acid phosphatase (TRAP) staining was used to determine the percentage of TRAP+ multinucleated cells in each group. In brief, the cells were washed three times with PBS and fixed in 4% paraformaldehyde (in PBS) for 15 min. TRAP solution (Procell Life Science & Technology Co.) was added, and the cells were stained for 60 min. Images were captured using an inverted microscope, and multinucleated cells were manually counted for analysis.

### *In vitro* osteoblastic differentiation

MEFs (5×10^3^ per well) were seeded into 24-well plates and cultured overnight. Then, the culture medium was changed to osteogenic induction medium for the different treatment groups, namely, PBS (OBM group), PLGA, Mg(OH)_2_@PEG-PLGA, and 2Mg@PEG-PLGA, for 7 and 14 days. For alkaline phosphatase (ALP) staining, the cells were washed with PBS and fixed with 4% paraformaldehyde for 15 min. Then, the cells were washed three times with PBS and stained with a BCIP/NBT ALP color development kit (Beyotime, China). ALP activity was assessed via an ALP assay kit (Beyotime, China) according to the manufacturer's instructions. For alizarin red S (ARS) staining, cells were fixed as described above and stained with alizarin red working solution (Beyotime, China) for 30 min. To quantitatively analyze the results of ARS staining, 500 μL of 10% acetic acid was added to each well, and the absorbance was read at 405 nm with a plate reader. Cells treated with culture medium alone were used as the control group.

### Ultrasound-guided minimally invasive implantation of the Mg@PEG-PLGA hydrogel

To evaluate the feasibility of using the Mg@PEG-PLGA hydrogel combined with the ultrasound guidance for the minimally invasive treatment of bone defects, a rabbit model of femoral condylar defects (5 mm in diameter, 3 mm in depth) was established using a low-speed spherical grinding drill. Then, ultrasound-guided Mg@PEG-PLGA hydrogel (approximately 25 μL) injection was performed using an ultrasound machine (VisualSonics, Inc., Canada). Following a 5-min local infusion of 0.9% saline, the ultrasound images of the implant site were acquired.

### Osteoporotic rat bone defect model

All animal procedures adhered to the Guidelines of the Ministry of Science and Technology of Health Guide for Care and Use of Laboratory Animals, China, and received approval from the institutional ethics committee (IEC) of Chongqing Medical University. Female Sprague‒Dawley rats aged 10 weeks (250-300 g) underwent ovariectomy (OVX) and were then maintained for 12 weeks to establish an ovariectomized osteoporotic model. Subsequently, the femurs and vertebral bodies of some of the OVX rats were harvested, fixed in 4% paraformaldehyde for 48 h, and subjected to micro-CT (µCT100, Scanco Medical, Switzerland) and histological analysis. Micro-CT data analysis and histological staining of the distal femoral metaphysis were performed to evaluate the degree of bone loss in the OVX rats.

Other OVX rats were anesthetized with pentobarbital (40 mg/kg) via intraperitoneal injection. A cylindrical defect (3 mm for both diameter and depth) was created on the lateral epicondyle of the femur using a low-speed spherical grinding drill under continuous saline irrigation. The rats were randomly divided into four groups: (1) the defects were left unfilled (control group); (2) the defects were filled with the PLGA hydrogel (PLGA group); (3) the defects were filled with the Mg(OH)_2_@PEG-PLGA hydrogel (Mg(OH)_2_@PEG-PLGA group); and (4) the defects were filled with the 2Mg@PEG-PLGA hydrogel (2Mg@PEG-PLGA group). Approximately 20 μL of the hydrogel was injected to completely fill the bone defect. Following a 5-min immersion of the hydrogels in 0.9% saline, the surgical sites were sutured layer by layer. All the operations were performed under sterile conditions.

### *In vivo* characterization of osteoporotic bone defect repair

At four and eight weeks postsurgery, three rats per group were sacrificed to assess the bone repair efficacy of the hydrogels. The harvested femurs were fixed in 4% paraformaldehyde for 48 h for subsequent analysis. The samples were subjected to scanning with a micro-CT system (µCT100, Scanco Medical, Switzerland), and 3D reconstruction of the regenerated bone was conducted using micro-CT system software. The bone tissue volume/total tissue volume (BV/TV) was quantitatively analyzed and compared to evaluate the extent of bone regeneration. To evaluate the *in vivo* antioxidant effect of the hydrogel, differences in reactive oxygen species (ROS) in the bone defect were measured at the fourth week using dihydroethidium (DHE) staining, as previously reported 51. Following decalcification, the samples were prepared for subsequent histological examination. Hematoxylin and eosin (HE), TRAP, and immunohistochemical staining for osteopontin (OPN) and osteocalcin (OCN) were performed according to the manufacturer's instructions.

To further investigate the mechanisms underlying the promotion of osteoporotic bone defect repair by the 2Mg@PEG-PLGA hydrogel, we selected three rat femoral bone defect samples from each of the control and 2Mg@PEG-PLGA groups for transcriptome sequencing and subsequent bioinformatics analysis. Briefly, the samples were collected after 4 weeks of implantation, immediately immersed in liquid nitrogen, and stored at -80°C. DEGs, defined by a fold change > 2 and a P value < 0.05, were identified via one-way analysis of variance (ANOVA). Functional enrichment analysis of the DEGs was conducted using the Gene Ontology (GO) platform (http://www.geneontology.org/). Additionally, the Kyoto Encyclopedia of Genes and Genomes (KEGG; http://www.genome.jp/kegg/pathway.html) was used to identify pathways significantly associated with the DEGs. Pathways with P value thresholds < 0.05 were considered potential target pathways for further exploration.

### Statistical analysis

All the statistical analyses were performed using GraphPad Prism software 8.0.2. The data are expressed as the mean ± standard deviation (SD). The significance of the differences between groups was analyzed via one-way ANOVA (* *p* < 0.05, ** *p* < 0.01, *** *p* < 0.01 and # *p* < 0.05, ## *p* < 0.01, ### *p* < 0.001).

## Supplementary Material

Supplementary figures and table.

## Figures and Tables

**Table 1:** Composition of PLGA and Mg@PEG-PLGA hydrogels

Groups	Mg@PEG (mg)	PLGA hydrogel (mL)
PLGA	0	1
0.25Mg@PEG-PLGA	0.25	
0.5Mg@PEG-PLGA	0.5	
1Mg@PEG-PLGA	1.0	
2Mg@PEG-PLGA	2.0	
4Mg@PEG-PLGA	4.0	

## Abbreviations

ROS: reactive oxygen species
Mg: magnesium
H_2_: hydrogen
PEG: polyethylene glycol
PLGA: poly(lactic-co-glycolic acid)
PMMA: polymethylmethacrylate
•OH: hydroxyl radical
HA: hyaluronic acid
ISFIs: in situ forming implants
NMP: 1-methyl-2-pyrrolidinone
SEM: scanning electron microscopy
EDS: energy dispersive spectroscopy
XRD: X-ray diffraction
XPS: X-ray photoelectron spectroscopy
FTIR: Fourier transform infrared
PBS: phosphate buffer solution
MB: methylene blue
MEFs: mouse embryonic fibroblasts
DCFH-DA: 2',7'-dichlorofluorescin diacetate
TRAP: tartrate-resistant acid phosphatase
OCM: osteoclast inductive medium
OBM: osteoblastic induction medium
ALP: alkaline phosphatase
ARS: alizarin red S
OVX: ovariectomy
CT: computed tomography
ICP-OES: inductively coupled plasma optical emission spectrometer
DHE: dihydroethidium
IHC: immunohistochemical staining
OPN: osteopontin
OCN: osteocalcin
DEGs: differentially expressed genes
ALT: alanine aminotransferase
AST: aspartate aminotransferase
BUN: blood urea nitrogen
CREA: creatinine
