# Supplementary material for: An injectable magnesium-loaded hydrogel releases hydrogen to promote osteoporotic bone repair via ROS scavenging and immunomodulation
Source: Theranostics. 2024 Jun 17;14(9):3739–59. doi: 10.7150/thno.97412 (PMC11209720; doi:10.7150/thno.97412)
Supplement: Supplementary file 1 — Supplementary figures and table. [file thnov14p3739s1.pdf]

Supporting Information

An injectable magnesium-loaded hydrogel releases hydrogen to promote osteoporotic bone repair via ROS scavenging and immunomodulation

Hang Zhou[1,4], Zhongyuan He[1], Youde Cao[2], Lei Chu[1], Bing Liang[2,#], Kexiao Yu[3,#] Zhongliang Deng[1,#]

[1] Department of Orthopaedics, the Second Affiliated Hospital of Chongqing Medical University, 76 Linjiang Road, Yuzhong Distinct, Chongqing, 400010, P. R. China.

[2] Department of Pathology from College of Basic Medicine, and Molecular Medicine Diagnostic & Testing Center, and Department of Clinical Pathology Laboratory of Pathology Diagnostic Center, Chongqing Medical University, 1 Yixueyuan Road, Yuzhong Distinct, Chongqing 400016, P. R. China.

[3] Department of Orthopedics, Chongqing Traditional Chinese Medicine Hospital, The First Affiliated Hospital of Chongqing College of Traditional Chinese Medicine, No. 6 Panxi Seventh Branch Road, Jiangbei District, Chongqing 400021, P. R. China.

[4] Department of Ultrasound & Chongqing Key Laboratory of Ultrasound Molecular Imaging, the Second Affiliated Hospital of Chongqing Medical University, Chongqing 400010, P. R. China.

# Corresponding authors: doctorliang51@163.com (B. Liang), csyxk@126.com (K. Yu), dengzl@cqmu.edu.cn (Z. Deng)

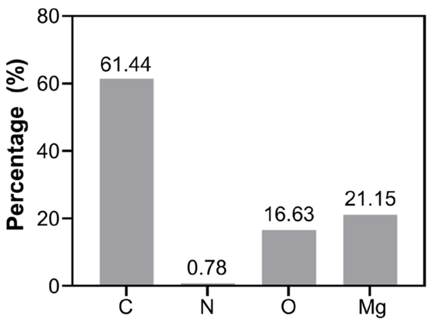

Figure S1. Elemental percentages of the Mg@PEG microparticles.

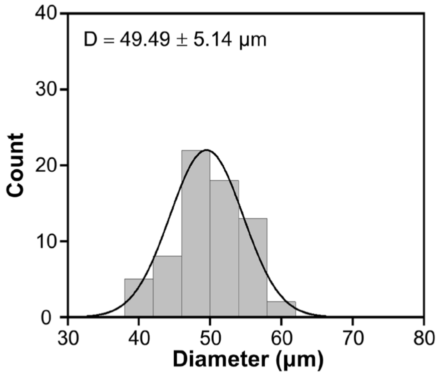

Figure S2. The size distribution of Mg particles.

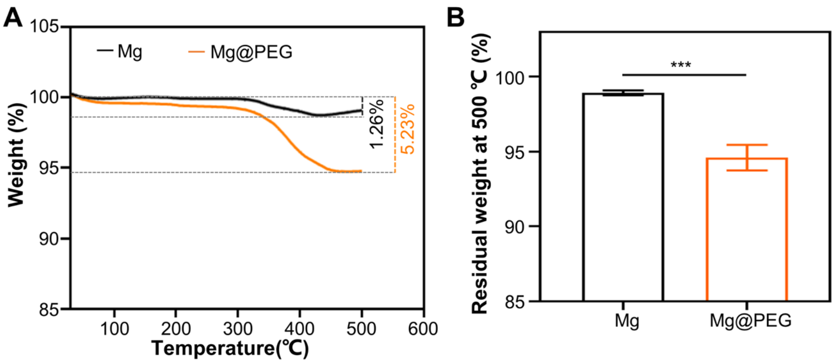

Figure S3. Thermogravimetric analysis of Mg and Mg@PEG particles. $***p < 0.001$

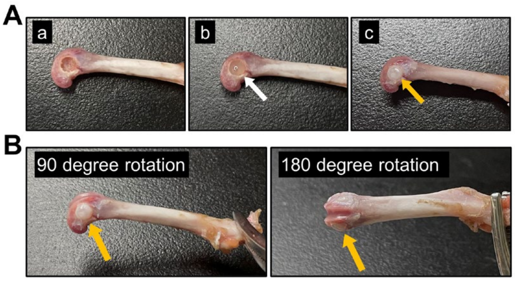

Figure S4. The ability of the Mg@PEG-PLGA hydrogel to bind to bone defect sites. (A) Twenty microliters of the Mg@PEG-PLGA hydrogel (white arrow) was injected to completely fill the rat femoral condylar defect, and the hydrogel solidified after 5 min of irrigation with 0.9% saline (yellow

arrow). (B) No displacement or deformation of the solidified Mg@PEG-PLGA hydrogel (yellow arrow) was observed during the rotation test.

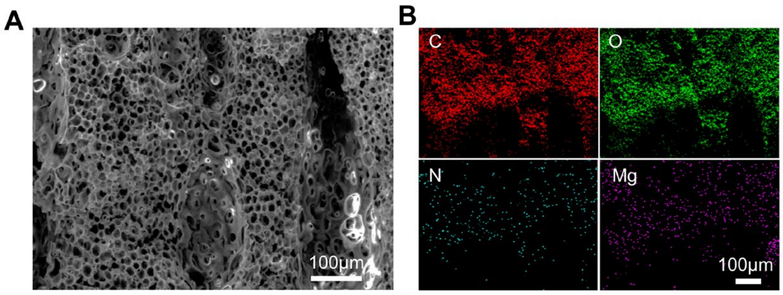

Figure S5. (A) SEM images and corresponding (B) element mapping images of the solidified Mg@PEG-PLGA gel.

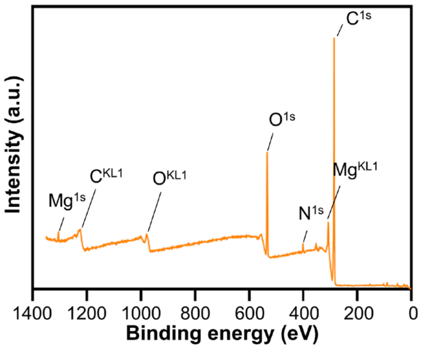

Figure S6. XPS spectra of the solidified Mg@PEG-PLGA gel.

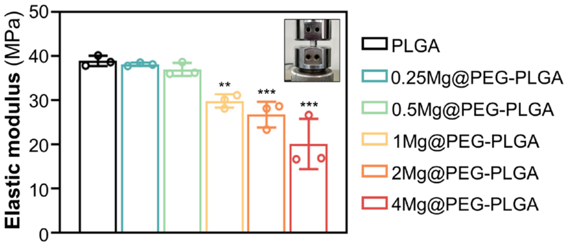

Figure S7. The elastic modulus of the solidified PLGA and Mg@PEG-PLGA gels (loaded with different Mg contents). Insert figure shows the mechanical test of the solidified hydrogel. **$p < 0.01$ and ***$p < 0.001$

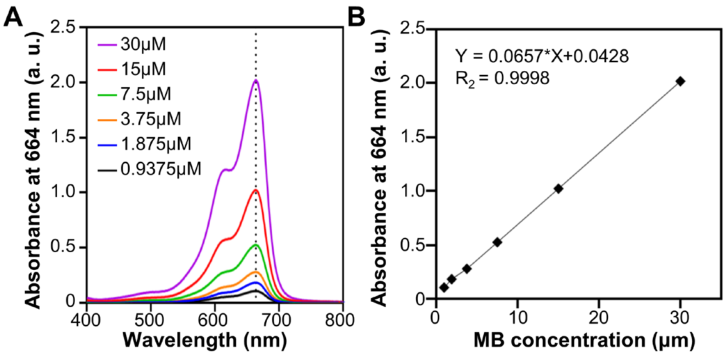

Figure S8. (A) Absorption spectra of MB at different concentrations; (B) the standard curve of absorbance vs MB concentration.

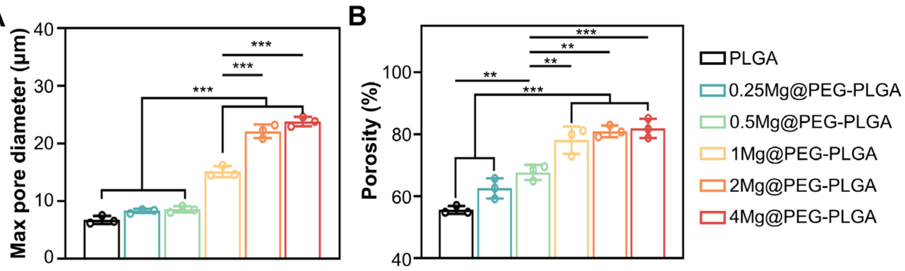

Figure S9. The (A) max pore diameter analysis of the solidified PLGA and Mg@PEG-PLGA gels measured by ImageJ. (B) Porosity test of the solidified PLGA and Mg@PEG-PLGA gels. **$p < 0.01$ and ***$p < 0.001$

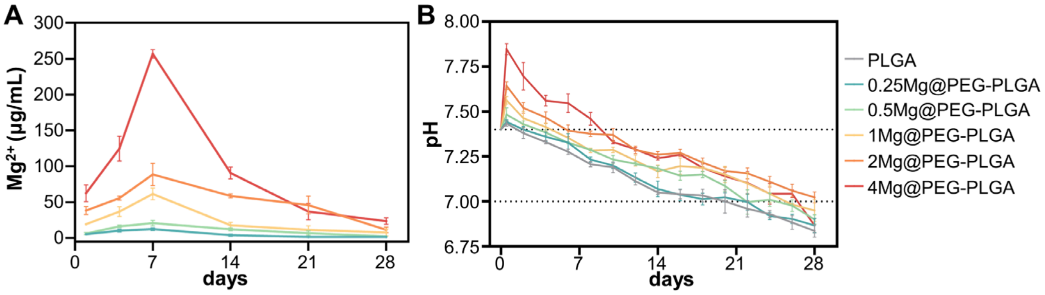

Figure S10. Degradation studies of PLGA and Mg@PEG-PLGA hydrogels. (A) The Mg^{2+} ions release behavior of different Mg@PEG-PLGA hydrogels. (B) Changes in the pH of PBS solution with immersed PLGA or Mg@PEG-PLGA hydrogels.

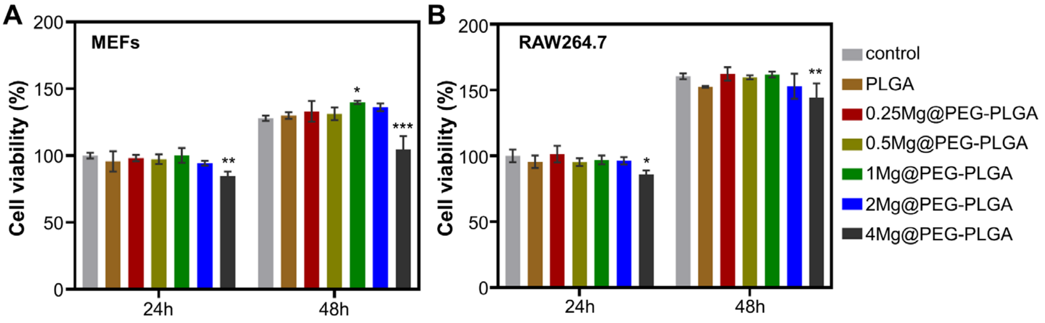

Figure S11. CCK-8 assay of the (A) MEFs and (B) Raw264.7 cells cultured with or without hydrogels. *$p < 0.05$, **$p < 0.01$ and ***$p < 0.001$, compared with the control group.

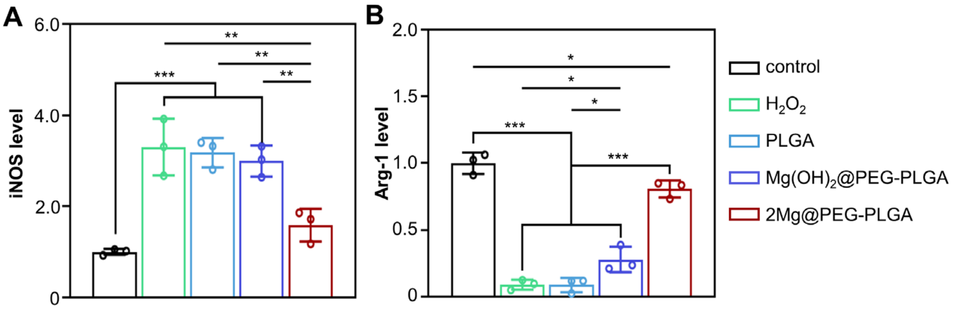

Figure S12. The relative fluorescence intensity quantification of iNOS and Arg-1. *$p < 0.05$, **$p < 0.01$ and ***$p < 0.001$

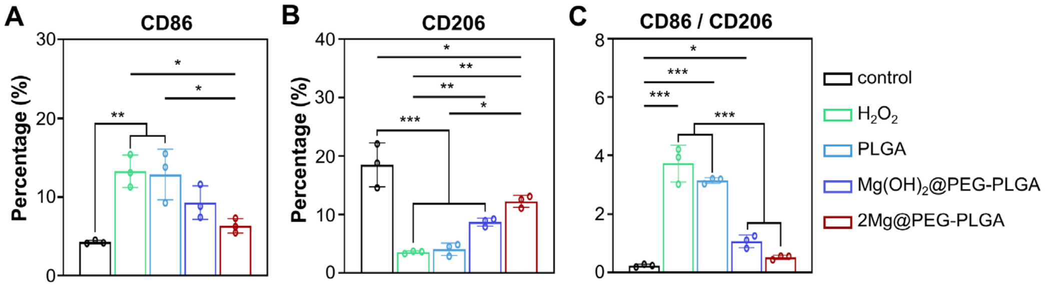

Figure S13. Quantification of the percentages of (A) CD86+ and (B) CD206+ macrophages and (C) the ratio of M1/M2 (CD86+/CD206+) macrophages. *$p < 0.05$, **$p < 0.01$ and ***$p < 0.001$

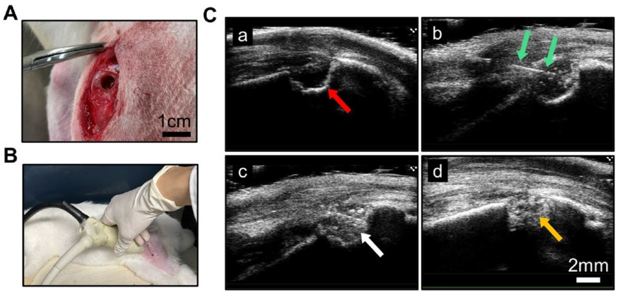

Figure S14. Ultrasound-guided minimally invasive implantation of the Mg@PEG-PLGA hydrogel. (A) A rabbit model of femoral condylar defects (5 mm in diameter, 3 mm in depth) was established.

(B) An ultrasound machine was used for the local percutaneous injection of the Mg@PEG-PLGA hydrogel into the femoral condylar defect. (C) After locating the femoral condylar defect (red arrow), a needle (16 G) (green arrow) was slowly inserted into the defect site under continuous ultrasound guidance. Approximately 25 μL of the Mg@PEG-PLGA hydrogel (white arrow) was injected to completely fill the bone defect. Following a 5-minute local infusion of 0.9% saline, a bone defect filled with solidified Mg@PEG-PLGA hydrogel (yellow arrow) was observed.

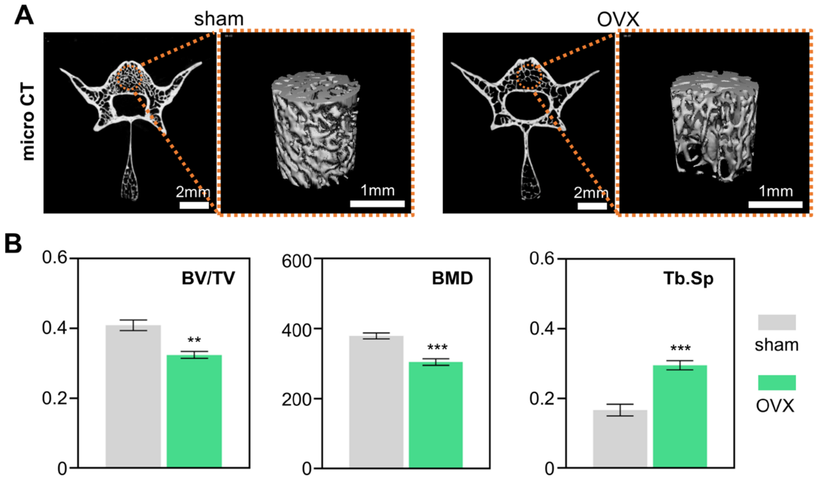

Figure S15. (A) The micro CT image of vertebral body and corresponding (B) quantitative analysis: (a) ratio of bone volume/total volume (BV/TV), (b) bone mineral density (BMD), and (c) trabecular separation (Tb.Sp).

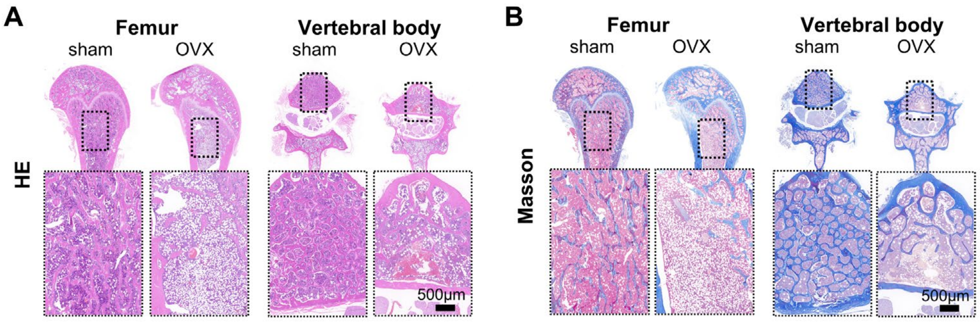

Figure S16. HE and Masson staining of the femurs and vertebral bodies in the sham and OVX groups.

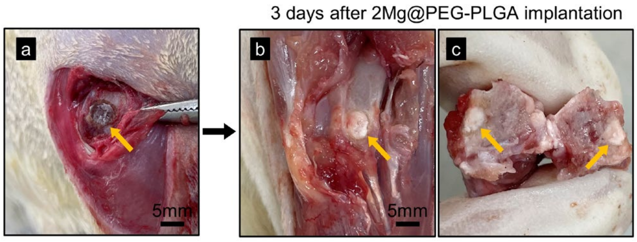

Figure S17. There was no observable material leakage into the surrounding tissue 3 days after

Mg@PEG-PLGA *in vivo* implantation.

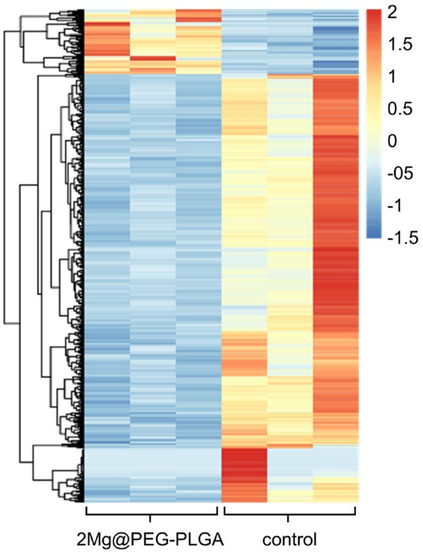

Figure S18. Heatmap representing the top 100 genes significantly differentially expressed between the 2Mg@PEG-PLGA group and the control group.

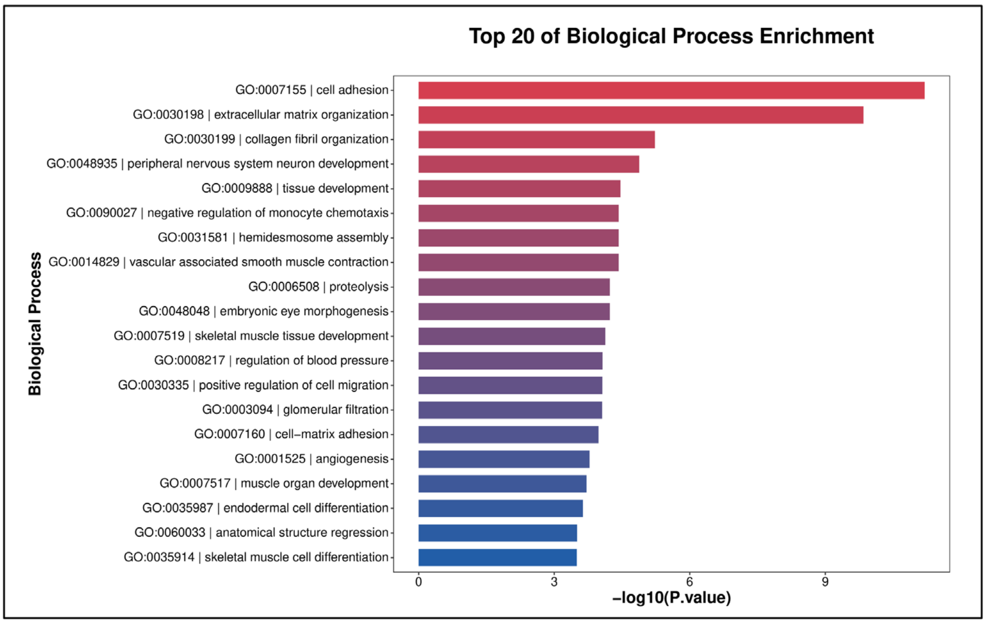

Figure S19. Functional enrichment analysis of the differentially expressed genes in 2Mg@PEG-PLGA gel treated rats as compared to the control group.

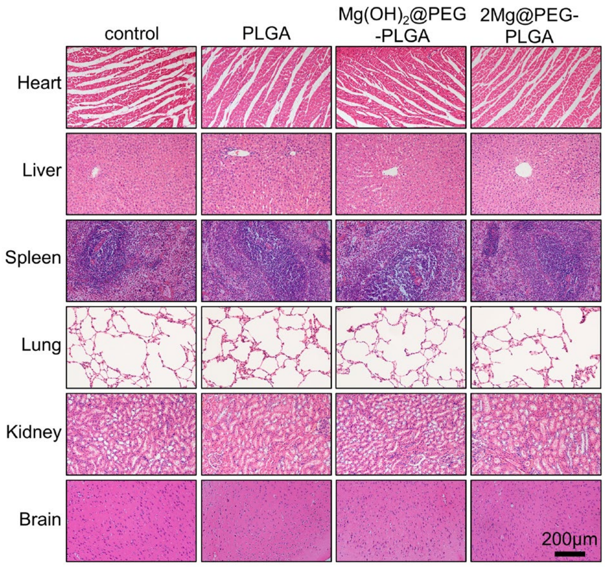

Figure S20. HE staining images of major organs of the rats in different groups.

Supporting table

Table S1. Serological analysis of the experimental rats.

	Reference ranges	Control (4W)	2Mg@PEG-PLGA (4W)	Control (8W)	2Mg@PEG-PLGA (8W)
**ALT** U/L	73.69 ± 24.12	72.26 ± 14.28	80.65 ± 6.39	81.60 ± 10.20	78.65 ± 9.51
**AST** U/L	173.32 ± 43.75	150.21 ± 18.95	169.64 ± 23.21	170.21 ± 30.27	162.71 ± 10.84
**BUN** mg/dl	20.33 ± 3.99	21.02 ± 0.95	19.84 ± 1.02	19.96 ± 2.01	21.65 ± 0.82
**CREA** μmol/L	47.25 ± 16.51	43.54 ± 4.54	45.84 ± 9.21	48.37 ± 8.54	45.89 ± 6.02
**Mg^{2+}** mmol/L	0.67-1.85	1.15 ± 0.21	1.28 ± 0.20	0.95 ± 0.18	1.24 ± 0. 36
